# Mpox-specific cellular and humoral immunity in mpox survivors living with HIV

**DOI:** 10.1016/j.celrep.2025.116501

**Published:** 2025-10-31

**Authors:** Samuel D. Stampfer, Lalita Priyamvada, Shainy Sambyal, Sailaja Gangadhara, Margaret Moriarty, Panayampalli S. Satheshkumar, Alba Grifoni, Alessandro Sette, Anandi N. Sheth, Colleen F. Kelley, Rama R. Amara

**Affiliations:** 1 Division of Microbiology and Immunology, Emory Vaccine Center, Emory National Primate Research Center, Emory University, Atlanta, GA, USA; 2 Department of Microbiology and Immunology, Emory School of Medicine, Emory University, Atlanta, GA, USA; 3 Division of Infectious Diseases, Department of Medicine, Emory School of Medicine, Emory University, Atlanta, GA, USA; 4 Poxvirus and Rabies Branch, Centers for Disease Control and Prevention, Atlanta, GA, USA; 5 Center for Infectious Disease and Vaccine Research, La Jolla Institute for Immunology (LJI), La Jolla, CA, USA; 6 Department of Medicine, Division of Infectious Diseases and Global Public Health, University of California, San Diego, La Jolla, CA, USA; 7 Grady Health System, Atlanta, GA, USA; 8 Lead contact

## Abstract

The 2022 mpox virus (MPXV) outbreak caused life-threatening mpox disease in people living with HIV (PLWH). We compared immune responses to MPXV in PLWH who were either mpox-naive or were mpox survivors. We characterized the magnitude, polyfunctionality, and cytolytic potential of CD4 and CD8 T cells specific to MPXV and cross-reactive orthopoxviral antigens and compared them to HIV− and cytomegalovirus (CMV)-specific T cells. Mpox survivors developed MPXV-specific CD4 T cells capable of producing IFNγ, TNFα, and IL-2. Their polyfunctionality was superior to CMV-specific CD4 T cells. MPXV- and CMV-specific CD8 T cells had similar cytolytic potentials. Importantly, mpox survivors developed strong MPXV-specific antibodies with neutralizing activity. Prior vaccinia or JYNNEOS vaccination was associated with greater MPXV-specific antibody but not T cells. These data demonstrate that PLWH generate functional cellular and humoral immunity to MPXV post-mpox. Despite their immunocompromised state, mpox survivors with HIV are expected to have some protection against future MPXV exposures.

## In brief

Mpox virus (MPXV) causes life-threatening mpox disease in HIV-positive individuals. Stampfer et al. evaluated 15 HIV-positive mpox survivors and detected polyfunctional MPXV-specific CD4 and CD8 immunity along with strong binding and neutralizing antibodies. These were even stronger in the 7/15 patient subsets with prior smallpox vaccination who also had broader immunity.

## Graphical Abstract

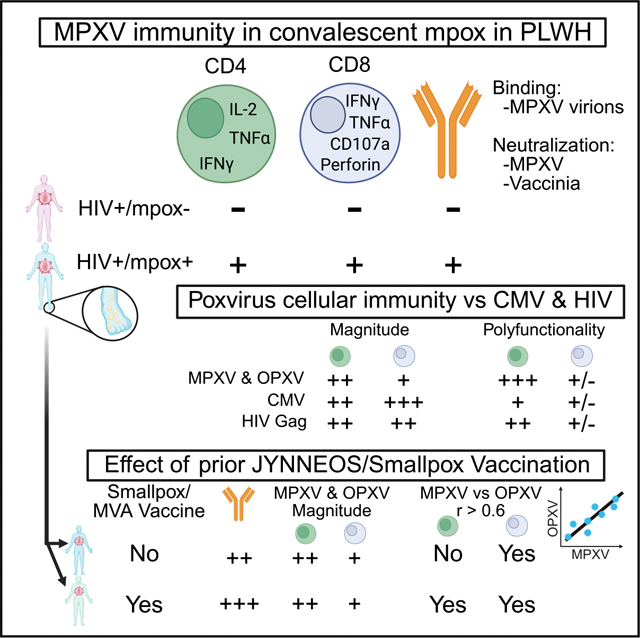

## INTRODUCTION

Mpox virus (MPXV) is an orthopoxvirus that has caused periodic outbreaks of mpox disease over the past 50+ years.^1^ It is related to variola (the causative agent for smallpox) and vaccinia (VV; smallpox vaccine) viruses. Unlike smallpox, which only affects humans,^2^ mpox is a zoonosis with an animal reservoir thought to be rodents in Africa, where most outbreaks have originated. Previously, mpox spread was limited by inefficient human-to-human transmission along with pre-existing population immunity conferred by smallpox vaccination.^1,3^ In 2022, a large mpox outbreak of the newly recognized subclade IIb resulted in over 95,000 documented infections worldwide, primarily due to human-to-human transmission via sexual contact. Most cases were mild, featuring fever, malaise, and clusters of classic orthopoxvirus pustular skin lesions.^4^ Some severe cases occurred, primarily in immunocompromised patients, resulting in an overall 0.2% death rate.^4^ Severe mpox features include necrotic lesions on skin and organs that can result in digit amputation, tracheal obstruction necessitating intubation, organ dysfunction, bacterial superinfection, and septic shock complicated by multi-organ failure.^5–8^ More recently, a large outbreak of the deadlier clade I MPXV started in 2023 in the Democratic Republic of the Congo and has since spread outside of Africa, with over 55,000 laboratory-confirmed cases and increased spread partly due to sexual transmission.^4,9,10^ These two ongoing outbreaks highlight the increasing pandemic potential of MPXV.

A replication-competent VV vaccine, Dryvax, was used to eradicate smallpox. Others and we have showed that a single dose of Dryvax can induce strong VV-specific T cells and neutralizing antibodies that persist for greater than 80 years.^11–13^ Dryvax induces cross-reactive immunity against MPXV and provides protection against mpox in rhesus macaques.^14–16^ Nonhuman primate (NHP) studies showed that a strong antibody response to mpox is critical for this protection.^17^ The exact correlates of protection for mpox and smallpox in humans are not known,^18^ although neutralizing antibody titers ≥ 1:32 conferred 100% protection against smallpox in a prospective cohort of 127 exposed individuals (with 3/15 individuals who had titers < 1:32 developing infections).^19^ The US stopped vaccinating the general population for smallpox in 1972, due both to its eradication in the US as well as adverse effects in some populations, particularly immunocompromised individuals and those with eczema.^20^ More recently, live, non-replicating modified VV Ankara (MVA)-based vaccines have been developed for use both as viral vector vaccines for other pathogens as well as for smallpox and mpox.^21–23^ We and others showed that two doses of MVA induce cross-reactive MPXV-specific immunity and protect from mpox disease in NHPs.^14–16^ JYNNEOS (Imvanex) is the only currently approved MVA-based vaccine and is used to prevent smallpox and mpox. In the 2022 mpox epidemic, it was 86% effective in the general population following two doses of vaccine.^24^

In addition to the magnitude (frequency), the polyfunctionality (defined by co-expression of multiple cytokines) of T cells is critical for protection against infectious diseases. Others and we previously demonstrated that polyfunctional T cells are superior to monofunctional T cells by producing higher amounts of cytokine per cell^25^ and are associated with superior HIV control.^26,27^ People living with HIV (PLWH) are at uniquely high risk for both exposure to mpox and severe disease. Many are men who have sex with men (MSM), the major demographic affected by mpox in the 2022 epidemic.^28^ HIV results in higher risk both for reduced response to vaccinations^29^ and for developing severe mpox.^6,8,30^ In a large Atlanta-based cohort of mpox patients, HIV positivity conferred a 2.5-fold increased risk of developing severe mpox. Among those patients, there was an additional 2.1-fold risk of severe mpox if patients had HIV viral loads greater than 200 copies/mL, with lower CD4 counts also associated with severe mpox.^30^ Another series of 382 mpox cases in PLWH found that all 27 deaths occurred in patients with CD4 counts below 200 cells/mm, with the majority in patients with CD4 < 100 cells/mm.^6^ Defective cell-mediated immunity likely plays an important role, as T cell responses to orthopoxviruses are critical for controlling infection within individual lesions and reducing overall symptom severity.^31–33^ For these reasons, we were particularly interested in evaluating immune responses, including polyfunctionality, in PLWH following MPXV infection.

In this work, we recruited a cohort of 15 individuals with HIV who had recent mpox infections. As controls, we obtained peripheral blood mononuclear cells (PBMCs) and sera from a cohort of 10 individuals with HIV and without mpox infection and with low likelihood of prior smallpox vaccination. We used an optimized intracellular cytokine staining (ICS) assay to evaluate the magnitude and quality of their cellular responses to mpox and to closely related orthopoxviruses and compared them to their responses to two chronic viral infections: HIV and cytomegalovirus (CMV). We additionally evaluated their direct humoral responses to mpox as well as cross-reactive immune responses to MVA. Our data show that, following MPXV infection, PLWH generate MPXV-specific CD4 T cells with polyfunctionality and CD8 T cells with cytolytic potential. The functional quality of mpox-specific CD4 T cells was superior to that of CMV-specific T cells, and the functional quality of MPXV-specific CD8 T cells was comparable to that of HIV− and CMV-specific T cells. The infected individuals also generated binding and neutralizing antibodies to MPXV. This work helps to improve our understanding of mpox infection-induced immunity in a uniquely vulnerable group such as PLWH.

## RESULTS

### PLWH induce strong CD4 and CD8 T cell responses following mpox

15 patients with long-standing HIV and recent MPXV infection (median 57 days post symptoms, interquartile range [IQR] 47–122 days, range 2–211 days) donated PBMCs and sera (Table 1). Mpox was mild to moderate in all cases but one who was hospitalized for 5 days at the beginning of his clinical course (Table S1). To ensure that our assays were detecting true orthopoxviral immunity in this immunocompromised population, our control group consisted of archived frozen PBMCs and sera from PLWH that were taken prior to the mpox epidemic, from individuals born after 1972 and thus too young to have received routine smallpox vaccination. Patients were all male and 93% African American. Availability of controls was limited to those who had sufficient quantities of PBMCs, resulting in slightly uneven matching (70% African American and 70% male). HLA typing was not performed due to the concern that the small sample size would preclude any HLA-specific conclusions. We utilized an ICS assay to detect orthopoxvirus-specific T cell responses by stimulating cells with peptide pools consisting of selected CD4 and CD8 epitopes derived from MPXV proteins (one MpoxCD4 and five MpoxCD8 pools optimized for CD4 and CD8 T cells, respectively), cross-reactive orthopoxviral epitopes with high conservation in VV and variola virus (OpoxCD4 and OpoxCD8),^34^ and live replication-attenuated MVA virus.

**Figure 1.**
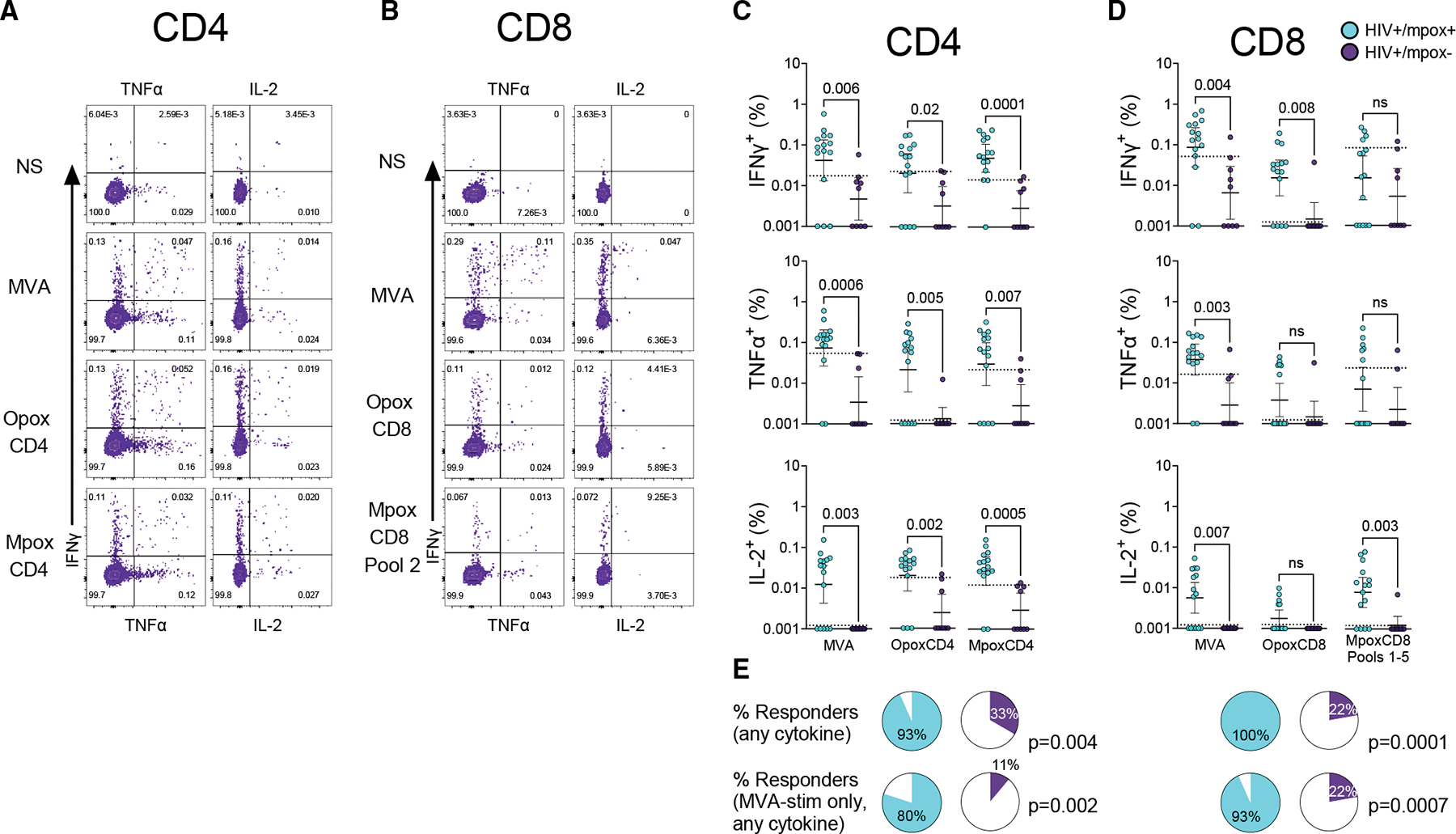
ICS analysis of mpox patients vs. controls Frozen PBMCs from HIV-positive individuals with recent mpox (*n* = 15; light blue) or without mpox (*n* = 9; purple) were thawed and then stimulated with poxvirus peptide pools (MPXV-specific MpoxCD4 and MpoxCD8 pools 1–5; cross-reactive orthopoxvirus OpoxCD4 and OpoxCD8 pools) or with 5 MOI of replication-deficient modified vaccinia Ankara (MVA). Signal was calculated by subtracting NS (non-stimulated) proportions from the stimulation condition. For MpoxCD8, pools 1–5 were done as 5 separate stimulations with the signal from each pool added together to determine the total CD8 signal. (A and B) Example intracellular cytokine stimulation (ICS) experimental results for CD4 (A) and CD8 (B) T cells. (C and D) Response rates to IFNγ, TNFα, and IL-2 are shown for CD4 (A) and CD8 (B) T cells. *p* values were calculated using Mann-Whitney tests. Data are represented as the geometric mean (solid line) and its 95% confidence interval (error bars). Dotted lines indicate the 90^th^ percentile cutoff of controls. (E) Assessment of overall CD4 and CD8 response rates. CD4 and CD8 responders were defined as those whose signal was above the 90th percentile of the controls for at least one cytokine in at least one stimulation condition. Fisher’s exact test (chi-squared) was used to calculate the significance of responder proportions in the patient group vs. the control group. See also Figures S1 and S2.

The MPXV and conserved orthopoxvirus peptide pools had been previously shown to detect T cell responses in individuals vaccinated for smallpox.^34^ Initial T cell assays were unable to detect strong signals above the background for peptide pools, but we were ultimately able to both improve the signal and reduce the background by utilizing a 16-h stimulation time, reducing the quantity of prestaining antibodies, and excluding cells with high side-scatter during flow cytometry. We improved the detection limit of the assay to below 0.01% for most specimens and cytokines by collecting the entire specimen and discarding the last few seconds of data (which have aberrant PE and PE derivative signals, possibly due to air entering the flow cytometer; Figure S1). Ultimately, we detected antigen-specific CD4 or CD8 T cell responses in all patients, comprising 0.01%–0.3% of the parent population when stimulated with peptide pools (Figures 1 and S2A). Stimulating with MVA yielded even stronger signals. The low-background assay resulted in low or undetectable responses in controls. Using the top 90^th^ percentile of the controls as a cutoff (a technique used when the true positive signal is not known^35–37^) resulted in the detection of CD4 responses from 14/15 patients in at least one stimulation and CD8 responses from all 15 patients, compared to 3/9 and 2/9 control specimens, respectively (one control specimen lacked usable PBMCs and was excluded from T cell analyses). This gave an overall sensitivity of 97% and specificity of 72%. Utilizing the MVA T cell responses alone resulted in a sensitivity of 86% and specificity of 83%, providing a reasonable alternative for a single stimulation.

CD4 responses were Th1 type, with significant increases in IFNγ, TNFα, and IL-2 secretion without increases in IL-4 or IL-17 (Figure S2B). CD8 responses were predominantly IFNγ based but with significant TNFα and even IL-2-positive CD8 T cells. Interestingly, the frequencies of CD4 T cells were comparable between OpoxCD4 and MpoxCD4 peptide stimulations as well as MVA live virus stimulation, but the frequencies of CD8 T cells tended to be greater with MVA live virus stimulation compared to OpoxCD8 and MpoxCD8 peptide stimulation. These data demonstrate that, despite being immunocompromised, PLWH can generate orthopoxvirus-specific T cells following mpox infection.

### MPXV- and orthopoxvirus-specific CD4 T cells have greater polyfunctionality than CMV-specific CD4 T cells

**Figure 2.**
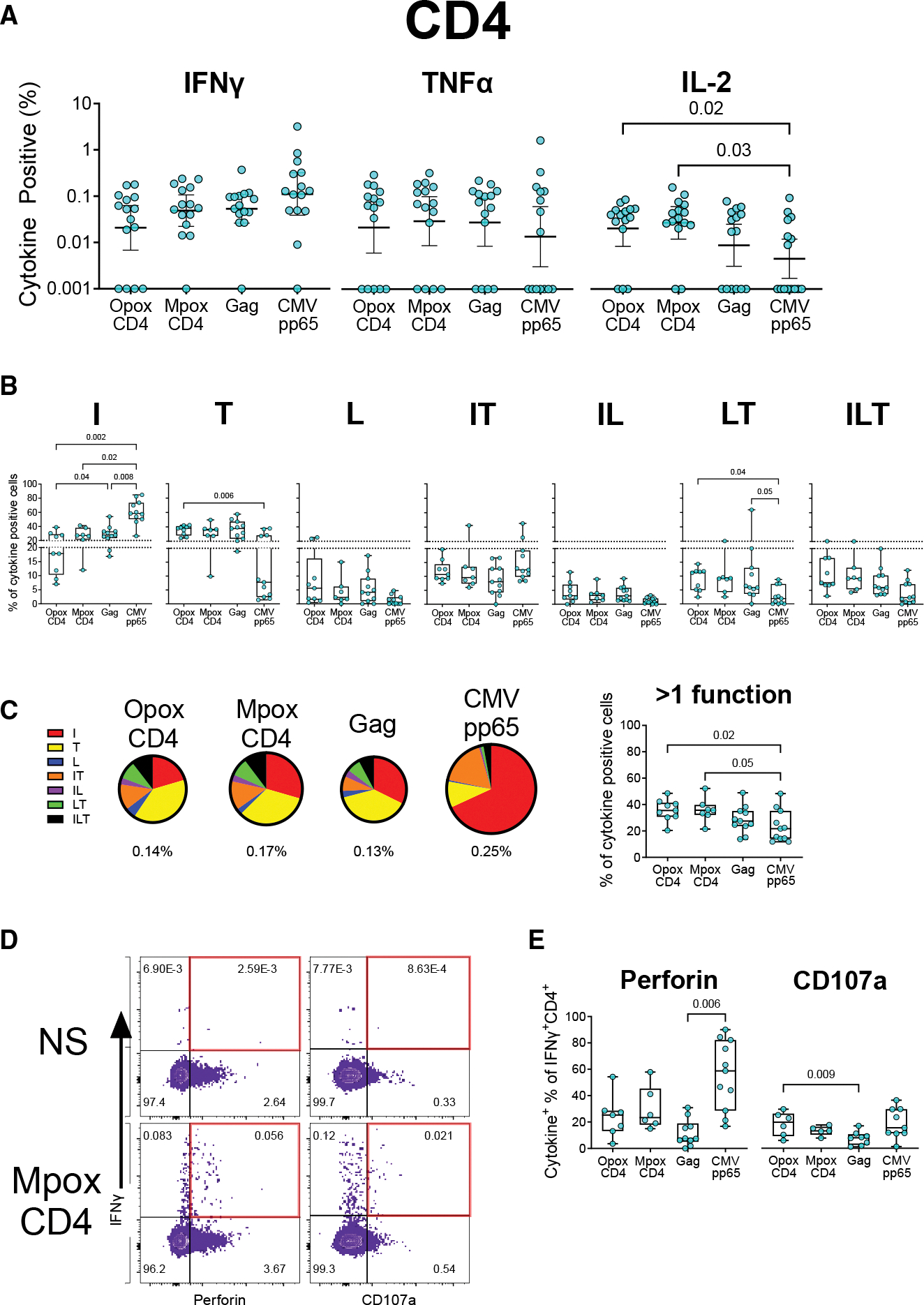
Comparison of CD4 responses to orthopoxviruses, CMV, and HIV Gag ICS was done on PBMCs, as described in Figure 1 (A) Proportions of cells positive for IFNγ, TNFα, and IL-2. Data are represented as the geometric mean (solid line) and its 95% confidence interval (error bars). (B and C) Proportions of cells positive for all combinations of IFNγ (I), TNFα (T), and IL-2 (L). The pie chart proportions (C) use the geometric means for each type of cytokine signal combination. Total signal is indicated underneath each pie chart with chart areas proportional to this signal. (D) Example flow plot for the evaluation of perforin and CD107a positivity as a proportion of all IFNγ-positive CD4 T cells. (E) Percent of IFNγ-positive cells with co-expression of either perforin or CD107a. *p* values were calculated using matched data using nonparametric tests in (A) (Friedman test) and parametric mixed-effects models in (B), (C), and (E). Multiple comparisons were corrected using Dunn’s test (A) and Tukey’s test (B, C, and E). Only samples with at least 20 cytokine-positive cells were included in polyfunctionality analyses (C, D, and E); geometric means for total signal in (C) are based on all samples. Data in box-and-whisker graphs in (B), (C), and (E) are represented as the median (solid line), with the box indicating 25^th^ to 75^th^ percentiles and whiskers extending to the maximum and minimum values. See also Figure S3.

To further understand the functional quality of MPXV-specific CD4 T cells, we determined the co-expression profiles for cytokines IFNγ, TNFα, and IL-2 using the Boolean function of Flowjo. We also evaluated cross-reactive orthopoxvirus T cells in the same manner. We compared both to specific responses to HIV (using a Gag peptide pool) and CMV (using a CMV-pp65 peptide pool), as all mpox survivors were positive for both chronic viral infections. MpoxCD4, OpoxCD4, and HIV Gag-specific CD4 T cells had responses of similar magnitudes for IFNγ, TNFα, and IL-2 (Figure 2A). The peptide pool from CMV, a virus known for inducing strong cellular immune responses,^38^ induced a nonsignificantly higher IFNγ response than the other peptide pools. It simultaneously induced lower IL-2 responses than both MpoxCD4 (*p* = 0.03) and OpoxCD4 (*p* = 0.02). CD4^+^ responses to orthopoxviruses and—to a lesser extent—Gag were polyfunctional with roughly similar proportions of single-positive IFNγ, TNFα, and IL-2, although Gag induced a significantly higher proportion of IFNγ+ single-positive cells as compared with the OpoxCD4 stimulation (Figure 2B). By contrast, the majority of the CMV response was single-positive IFNγ, occurring at a significantly higher proportion compared to all other stimuli. CMV induced a reduced proportion of single-positive TNFα CD4 cells as compared to all other stimuli, though this was only significant as compared with OpoxCD4 (*p* = 0.006). CMV also had lower proportions of polyfunctional CD4 T cells (>1 function) as compared to both OpoxCD4 (*p* = 0.02) and MpoxCD4 (*p* = 0.05; Figure 2C). The polyfunctionality differences were driven in part by IL-2, which was rarely detected without co-expression of IFNγ or TNFα or both and was present at lowest levels in CMV. We note that the observed differences for polyfunctionality between MPXV-specific CD4 T cells and HIV or CMV-specific T cells was in part due to the former being an acute infection and the latter two being chronic infections. Nevertheless, these data provide an opportunity to compare the immune responses against different viral infections within the same individual.

Although not common, previous studies demonstrated the expression of cytolytic molecules such as perforin and granzyme B by CD4 T cells.^39–41^ To understand the cytolytic potential of poxvirus, HIV, and CMV-specific CD4 T cells, we examined the expression of perforin and CD107a (a marker of degranulation) on IFNγ-positive CD4 cells (Figure 2D). The perforin+ and CD107+ CD4 T cells specific to all three viruses were clearly detectable albeit at low frequencies (Figure S3). CMV had the highest co-expression of perforin, though this difference was only significant as compared to Gag (*p* = 0.006; Figure 2E). Gag-specific CD4 T cells also had reduced proportional CD107a expression in their IFNγ+ cells (*p* = 0.009 as compared to OpoxCD4). OpoxCD4 and MpoxCD4 responses were similar to each other, as expected. Overall, these data demonstrate that the magnitude of the CD4 T cell response to orthopoxviruses is similar to that against HIV Gag but less than CMV. Partly due to increased IL-2 expression, the orthopoxvirus CD4 response is more polyfunctional than that against CMV.

### Fewer CD8 T cells respond to MPXV antigens than to CMV or HIV gag, but they possess strong cytolytic potential

**Figure 3.**
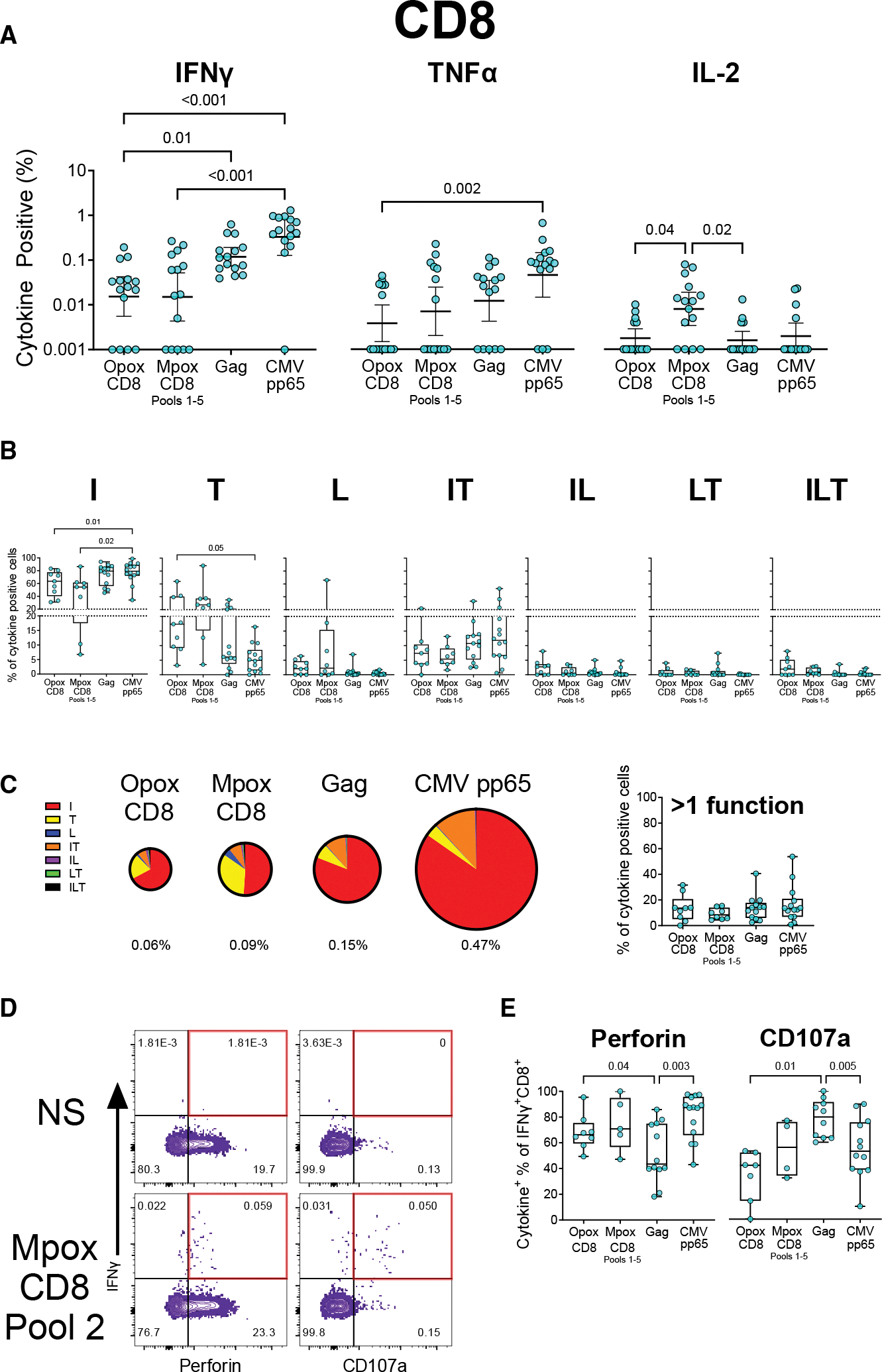
Comparison of CD8 responses to orthopoxviruses, CMV, and HIV Gag ICS was done on PBMCs, as described in Figure 1. (A) Proportions of cells positive for IFNγ, TNFα, and IL-2. Data are represented as the geometric mean (solid line) and its 95% confidence interval (error bars). (B and C) Proportions of cells positive for all combinations of IFNγ (I), TNFα (T), and IL-2 (L), with pie charts (C) based on the geometric means for each type and the relative percentages for each type. Total signal is indicated underneath each pie chart in (C) with chart areas proportional to this signal. Only samples with at least 20 cytokine-positive cells were included in polyfunctionality analysis, but total signal magnitude in (C) included all samples. (D) Example flow plot for the evaluation of perforin and CD107a positivity as a proportion of all IFNγ-positive CD8 T cells. (E) Percentage of all CD8+ IFNγ+ cells that expressed either perforin or CD107a (20 IFNγ-positive cells minimum). p values were calculated using matched data using nonparametric tests in (A) (Friedman test) and parametric mixed-effects models in (B), (C), and (E). Multiple comparisons were corrected using Dunn’s test (A) and Tukey’s test (B, C, and E). Data in box-and-whisker graphs in (B), (C), and (E) are represented as the median (solid line), with the box indicating 25th to 75th percentiles and whiskers extending to the maximum and minimum values. See also Figures S3, S4, S5, S6, S7, S8, and S9.

CD8 T cells were analyzed for virus-specific responses by ICS in a similar manner as CD4 (Figure 3A). One key difference, however, was that the MPXV-specific CD8 epitopes included 1647 distinct peptides, requiring their use in five separate MpoxCD8 peptide pools whose sum totals were later combined, in contrast to the single MpoxCD4 pool with 276 peptides.^34^ The conserved orthopoxvirus CD8 epitopes were better defined and used as a single pool of under 300 peptides (OpoxCD8).

Overall, the CD8 T cell response was predominantly IFNγ+, which was significantly weaker in both orthopoxvirus pools as compared to CMV (*p* < 0.0001 for OpoxCD8 and *p* = 0.0002 for mpox CD8; Figure 3A). OpoxCD8 also induced a significantly weaker IFNγ response as compared to Gag (*p* = 0.01). Similarly, the frequency of TNFα+ CD8 T cells tended to be lower in the OpoxCD8 (*p* = 0.002) and MpoxCD8 pools (*p* = 0.24), and the frequency of IL-2+ CD8 T cells was low across the board. The MpoxCD8 pool had the highest IL-2 expression, significantly higher than both Gag (*p* = 0.02) and OpoxCD8 (*p* = 0.04). The Boolean analysis revealed that the majority of CD8 T cell response consisted of single producers with higher proportional single IFNg+ and lower single TNFα + CD8 T cell responses for CMV-pp65-specific cells compared to OpoxCD8 (*p* = 0.01 for IFNγ+ and *p* = 0.05 for TNFα+)- and MpoxCD8 (*p* = 0.02 for IFNγ+ and *p* = 0.09 for TNFα+)-specific cells (Figure 3B). There was no significant difference in polyfunctionality between the peptide pools, which mostly featured single-positive or IFNγ^+^/TNFα^+^ cells (Figure 3C).

Next, we determined the cytolytic potential of CD8 T cells. As with the CD4 T cells, we analyzed perforin expression and degranulation (CD107a) on IFNg+ CD8 T cells (Figures 3E and S3). A significant fraction (median of 40%–80%) of OpoxCD8-, MpoxCD8-, Gag-, and CMV-specific IFNg+ CD8 T cells showed degranulation. Similarly, about 40%–90% of OpoxCD8, MpoxCD8, and CMV-specific IFNg+ CD8 T cells co-expressed perforin. However, the OpoxCD8-specific response was notable for having the lowest percentage of CD107a positivity among IFNγ-positive T cells, significantly lower than Gag (*p* = 0.01), which had the highest (Figure 3E). This effect was the opposite of that of perforin, where Gag-specific IFNγ-positive CD8 T cells were least likely to be perforin positive (*p* = 0.003 compared to CMV and *p* = 0.04 compared to OpoxCD8). Overall, these data demonstrated that the magnitude of IFNg+ MPXV-specific CD8 T cells is lower than Gag- and CMV-pp65-specific CD8 T cells. They also demonstrate that MPXV-specific CD8 T cells maintain a cytolytic potential similar to that of Gag- and CMV-specific CD8 T cells.

### PLWH mount strong binding and neutralizing antibody responses post-MPXV infection

**Figure 4.**
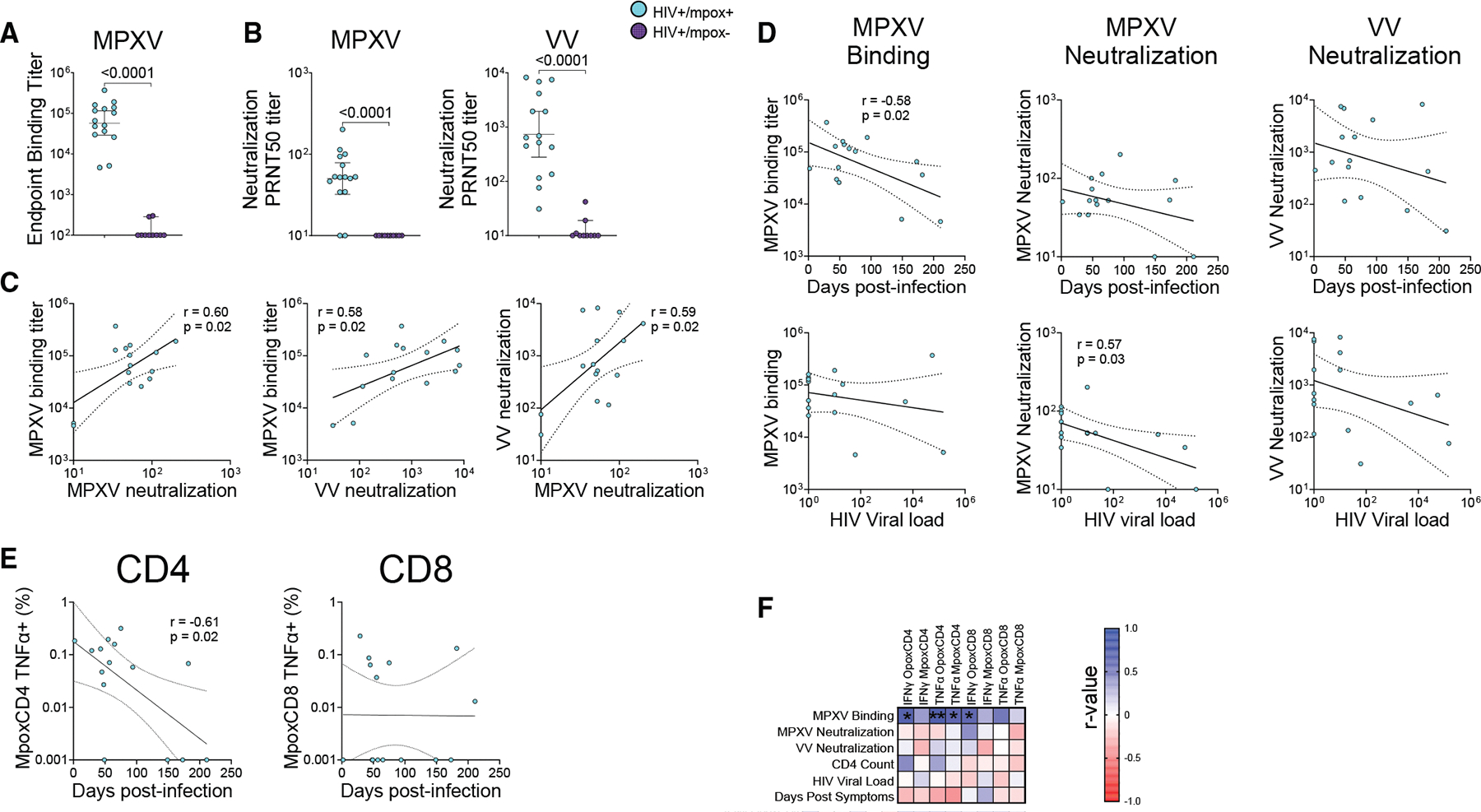
Humoral responses and temporal correlations in PLWH post mpox (A and B) Serologic samples from PLWH with recent mpox (*n* = 15) as well as PLWH without mpox (*n* = 10) were tested in a binding ELISA assay against inactivated MPXV (A) and in neutralization assays against live clade IIb MPXV and VV (B). (C and D) Endpoint titers of the mpox patients were log transformed and correlated by linear regression against each other (C) and against clinical parameters (days since their first symptoms of mpox infection or HIV viral load at time of infection) (D). (E) Days from first symptoms were also correlated by linear regression with the log-transform of T cell ICS TNFα signals. (F) A correlation matrix of humoral and clinical data vs. T cell parameters was generated nonparametrically using a Spearman correlation, with * denoting *p* < 0.05 and ** denoting *p* < 0.01. Data are represented by the geometric mean and 95% confidence interval in (A) and (B). All pairwise comparisons (A and B) were via the nonparametric Mann-Whitney *t* test. The 95% confidence bands of the best fit line are indicated by dotted lines (C, D, and E). See also Figure S4.

Patient and control sera were collected contemporaneously with their T cells. To measure the binding antibody response against MPXV, we developed a whole-virus binding ELISA assay, which was designed to capture the full breadth of anti-MPXV antibodies. This assay accurately distinguished between patients and unexposed controls. All patients and no controls had detectable antibodies to MPXV, and geometric mean titers were 2.7 logs higher in patients compared to controls (Figure 4A). The weakest patient titer of 1:4,600 was still 1.2 logs higher than the strongest control titer. We next evaluated MPXV (clade IIb) and VV neutralizing titers, which were measured as described previously.^42^ None of the controls had any detectable MPXV neutralization titers, and their VV titers were allbelow 1:50. Meanwhile, 13/15 patients had detectable MPXV neutralization titers, and 14/15 had VV titers >1:50 (Figure 4B). The lowest measured VV titer in an mpox patient was 1:31, which is close to the reported smallpox correlate of protection neutralizing titer of 1:32.^19^ This patient had an undetectable MPXV neutralization titer. The two patients with the weakest MPXV-binding titers also had the weakest VV neutralization titers and undetectable MPXV neutralization titers. The antibody data were log transformed and analyzed by linear regression. MPXV-binding and both MPXV and VV neutralization titers all correlated, with *p* values of 0.02 and r values of 0.58–0.60 (Figure 4C).

Antibody titers were lower in patients whose samples were obtained later with respect to mpox symptom onset. We observed a significant inverse correlation between sampling time and MPXV-binding titers (r = −0.58; *p* = 0.02; Figure 4D). However, there was no significant inverse association between MPXV or VV neutralization titer and time. HIV-control-associated clinical parameters (patient CD4 count and HIV viral load at time of sample collection) correlated strongest with MPXV neutralization titers. Higher HIV viral loads had a significant negative correlation with titers (r = −0.57, *p* = 0.03), while higher CD4 counts had a weaker, nonsignificant positive correlation (r = 0.45, *p* = 0.09) (Figures 4D and S4). In contrast, CD4 count had no association with MPXV-binding or VV neutralization titers, and HIV viral load had weaker negative correlations (r = −0.29 for MPXV binding and −0.39 for VV neutralization).

We also observed lower CD4 T cell cytokine positivity in samples obtained later, with significant negative correlations for OpoxCD4-specific IFNγ^+^ (r = −0.53, *p* = 0.04) and MpoxCD4-specific TNFα^+^ (r = −0.61, *p* = 0.02) as analyzed by linear regression (Figure 4E). Similar associations were noted when using nonparametric tests but did not reach statistical significance (Figure 4F). However, CD8 cytokine positivity did not correlate with sample acquisition date. Most T cell parameters had weak positive associations with binding and neutralization titers, but the MPXV-binding titer was the only antibody parameter that had statistically significant associations with T cell parameters, including both IFNγ and TNFα in CD4 T cells and IFNγ alone in CD8 T cells (Figure 4F). Interestingly, HIV VL and CD4 counts did not correlate with poxvirus-specific cellular immunity.

### Prior VV vaccination leads to stronger MPXV-specific antibody responses but not T cell responses post MPXV infection

In the US, childhood vaccination for smallpox was halted in 1972. Since then, VV-based vaccination has been reserved for those at highest risk of orthopoxvirus exposure. This has previously included members of the military and, since 2022, included individuals thought to be at increased risk for mpox. Thus, some patients in this study had exposure to both VV and MPXV, leading to hybrid immunity in 7/15 patients. Of these, three were born before 1972 and thus presumed to be vaccinated for smallpox, two received a dose of the JYNNEOS vaccine prior to acquiring mpox, and two additional patients were both born before 1972 and received JYNNEOS (one got a single dose prior to infection; the other received two doses post-infection but prior to sampling). No patients in the study received a full two-dose course of JYNNEOS prior to mpox, and none were in the military.

**Figure 5.**
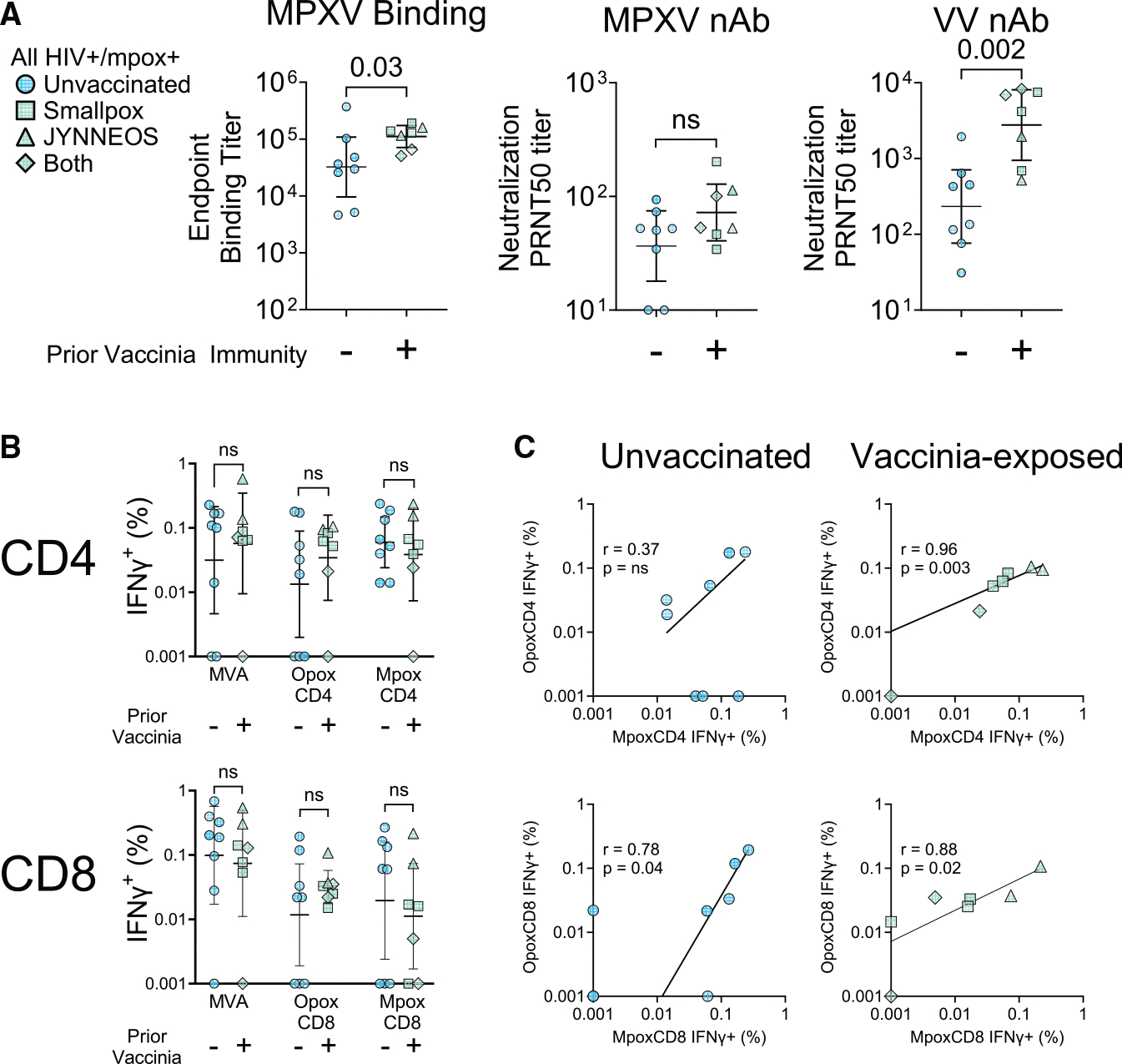
Comparison between subjects with and without prior smallpox vaccination (A and B) Patients were divided by a history of prior smallpox vaccination. Eight mpox survivors had no prior smallpox vaccination (circles), while three had historical pre-1972 smallpox vaccination (squares), two had a single dose of the JYNNEOS MVA vaccine (triangles; administered shortly before patients contracted mpox), and two had both historical smallpox and JYNNEOS vaccinations (diamonds). These two groups were compared with respect to antibody-binding titers and neutralization titers (nAb) (A) and orthopoxvirus-specific IFNγ+ CD4 and CD8 T cells (B); *p* values were calculated using Mann-Whitney tests. Data are represented by the geometric mean and 95% confidence intervals (A and B). (C) Within each group, we then correlated the T cell responses to MPXV-specific vs. cross-reactive orthopoxvirus-specific peptide pools in CD4 (top) and CD8 (bottom) T cells. R values and *p* values (95% two-tailed) were calculated using the nonparametric Spearman test. The best fit lines were placed using logarithmic nonlinear regression. See also Figures S5, S6, S7, and S8.

VV exposures had a strong influence on antibody titers. Vaccinated patients had significantly higher MPXV-binding titers than those exposed to MPXV alone (*p* = 0.03; Figure 5A). MPXV neutralization titers were not significantly different between groups, but VV neutralization titers were markedly higher in the pre-vaccinated group, with a geometric mean titer that was 1.1 logs higher than the unvaccinated group (*p* = 0.002; Figure 5A). Despite these differences, the group without VV exposure still had significantly higher MPXV-binding and both MPXV- and VV neutralization titers than the mpox-uninfected HIV-positive control group (Figure S5A). We did note that the prior exposure group had significantly lower HIV viral loads, which could also influence antibody formation, so we repeated the analysis by restricting it to subjects with HIV viral loads of <100. This included all seven vaccinated patients and 5/8 unvaccinated patients. In this subgroup with good virologic control, we still observed significantly higher MPXV-binding antibody titers (*p* = 0.01) and VV neutralization titers (*p* = 0.01) in patients with prior vaccination as compared with unvaccinated patients, indicating that prior vaccination does significantly influence antibody response to mpox infection (Figures S5B and S5C).

The enhanced VV neutralization effect was maintained for the three patients who were likely vaccinated for smallpox in childhood without any recent revaccination (indicated by squares; *p* = 0.02; Figure S6). This small group may have benefitted from MPXV infection, boosting their prior affinity-matured B cells dating back to their original smallpox vaccination, as well as optimal matching between the historical smallpox vaccine (vaccinia) and the VV used in the neutralization assays. Additionally, prior vaccination improved antibody durability. Unvaccinated individuals had stronger inverse correlations between time and antibody titers (r = −0.55 to −0.66) as compared to the vaccinia-exposed individuals (r = 0.04 to 0.38), suggesting that the combination of prior vaccination and mpox infection may promote longer-lasting antibodies than mpox infection alone (Figure S7).

Although humoral immunity was significantly stronger in the vaccinated individuals, we did not observe significant differences in the magnitude of poxvirus-specific T cell responses except for the CD8 TNFα response to MVA stimulation, which was lower in the prior-exposure group (Figures 5B and S8A). There were, however, subtle differences in the specificity of the cellular immunity between groups. The MPXV-specific CD4 T cells showed a strong correlation with orthopoxviral-specific CD4 T cells in the vaccinated group (r = 0.96; *p* = 0.003), and this association was not observed in the unvaccinated group, suggestive of the development of an MPXV-focused CD4 T cell response in the absence of prior exposure to cross-reactive orthopoxvirus T cell epitopes from VV (Figures 5C and S8B). However, for CD8 T cells, significant correlations were observed between MPXV- and orthopoxvirus-specific T cells in both unvaccinated (r = 0.78; *p* = 0.04) and prior-exposed (r = 0.88; *p* = 0.02) individuals, suggesting that the dominant MPXV CD8 T cell epitopes may be more cross-reactive than CD4. We additionally noted that unvaccinated individuals had a significantly higher percentage of IL-2-positive CD8 T cells compared to vaccinated individuals (Figure S9).

In summary, these data suggest that prior VV and single-dose JYNNEOS vaccination help with the generation of stronger MPXV-specific humoral immunity and more cross-reactive orthopoxviral-specific CD4 T cells following MPXV infection in PLWH.

## DISCUSSION

Mpox cases are spreading worldwide, and there is a great need to understand the protective immunity in infected people that will aid in developing effective vaccines. It is even more important to study immunity in PLWH who have compromised humoral and cellular immune systems. In this study, we characterized the mpox-specific immunity in PLWH who recovered from mpox. We focused our analyses on the magnitude and functional quality of mpox-specific humoral and cellular immunity and compared it with HIV− and CMV-specific immunity in the same individual.

Quantifying orthopoxvirus-specific cellular responses in mpox can be challenging, particularly in PLWH who tend to have lower CD4 T cell counts. Elispot assays have been used successfully but with conserved orthopoxvirus and MPXV-specific T cells detected typically at <0.05%^43,44^ of PBMCs. Meanwhile, others have used ICS and activation-induced marker (AIM) assays, which detected CD4 and CD8 T cell responses of 0.05%–1% but with high background in unexposed controls (over 50% in some assays).^34,45–47^ Other published assays either failed to detect CD8 T cell responses in a majority of patients by ICS^46^ or detected only weak CD4 and CD8 responses in nearly all patients.^48^ We saw a stronger response rate with lower background in our study, perhaps due to different stimulation conditions despite using the same peptide pools as some. In particular, we suspect that this was due to the 12-h peptide co-incubation stimulation time followed by 4 h of GolgiStop and GolgiPlug. Our modified ICS technique could be useful going forward for improved characterization of post-mpox cellular immunity in the ongoing clade I and IIb mpox outbreaks.

As an alternative, using our assay data for MVA stimulation alone resulted in lower background (1/9 positive controls for CD4 and 2/9 for CD8) while detecting CD8 T cell responses in 14/15 patients, though sensitivity was lower for CD4 (12/15 patients). MVA alone is thus an attractive option for screening for T cell responses in a larger cohort to reduce the number of samples per assay. It has the added advantage of being unbiased with respect to HLA as it presents entire protein products. In contrast, peptide pools used in the current and previous studies^34,46–48^ are limited to several hundred 8–20 amino acid epitopes that represent only a small fraction of sequences from the 190–200-kb VV and MPXV genomes and are selected based on their T cell immunodominance toward common HLA haplotypes in a population. They can thus occasionally fail to detect T cell responses that are detectable by MVA stimulation.^49^

We found that the magnitudes of the CD4 T cell responses to MPXV and conserved orthopoxviral epitopes were comparable to those to HIV Gag and CMV pp65. In contrast, the CD8 T cell magnitude was higher for both HIV Gag and CMV pp65 as compared with the orthopoxviruses. This might be because of better persistence of orthopoxvirus-specific CD4 T cell responses than CD8 T cell responses, as we observed previously following Dryvax vaccination in humans.^13^ We also noted greater polyfunctionality among orthopoxvirus-specific CD4 T cells as compared to Gag and CMV but did not note much qualitative differences in CD8 T cells. Based on this, we hypothesize that highly functional CD4 T cells may contribute significantly to eliminating MPXV infection. Heavier reliance on CD4 T cells (as opposed to CD8) could partly account for the much greater severity of mpox observed in people living with AIDS, who all have severely reduced CD4 T cell counts in the setting of reduced-to-normal CD8 T cell counts. Future studies comparing MPXV-specific CD4 T cell polyfunctionality in cases of mild vs. severe mpox could test this hypothesis.

Our low-background whole-virus MPXV-binding ELISA detected measurable serologic immunity among all mpox patients without any false positivity among the control cohort, with the weakest positive patient having a measured titer that was 1.2 logs higher than the strongest control patient. This performed better than a similar whole virus assay done on clade IIb convalescent patients^46^ and was comparable to serologic results from the 2003 US clade IIa mpox outbreak.^50^ A recent study showed that H3L and A35 proteins of MPXV can be used to detect MPXV-specific antibody responses following MVA-BN vaccination.^51^ However, the whole-virus ELISA has the advantage of capturing the full breadth of serologic immunity, which may be helpful to quantify antibody levels and detect prior exposure in patients with weaker serologic responses. We found that two of our patients had MXPV neutralization titers below the detection limit despite having detectable MPXV-binding titers and cellular immunity. In our cohort, poorer MPXV neutralization in mpox patients correlated with incomplete suppression of HIV. Interestingly, HIV viremia has also been associated with severe mpox.^30^ Together, these findings raise concern that this particularly immunocompromised population might remain susceptible to MPXV reinfection in the future by virtue of their lower MPXV neutralization titers. We hypothesize that they may be unlikely to be at risk for severe mpox, however, as all have detectable cellular and humoral immunity that would be further boosted at the time of reinfection.

We were fortunate that our mpox patient cohort was split roughly evenly between those with prior exposure to VV vaccines (7 patients) and those without (8 patients). Even with the small sample size, we were able to detect a better cross-reactive orthopoxvirus response in the prior exposure group as compared to the unvaccinated group. The prior-exposed group had significantly higher MPXV-binding titers and VV neutralization titers, consistent with an anamnestic response to VV-specific memory B cells. Even excluding the four patients with recent vaccination (three of whom received just one dose of JYNNEOS), we still detected a difference among patients whose only exposure was to childhood vaccination over 50 years prior. It is remarkable that this effect was maintained given their interval acquisition of HIV and subsequent immunodeficiency.

Additionally, prior VV and JYNNEOS exposure influenced CD4 T cell specificity more than CD8 T cell specificity with respect to cross-reactive orthopoxvirus-specific T cells. The total IFNγ+ CD4 T cell responses specific to MPXV correlated strongly with those to conserved orthopoxviruses in mpox patients with prior exposure but did not correlate in those without prior exposure. IFNγ+ CD8 T cell responses had more similar correlations between the groups. This demonstrates a strong influence of prior VV vaccination on CD4 T cell specificity, leading to better recall of cross-reactive T cells after mpox exposure. As discussed above, our previous work showed that Dryvax vaccinees have a higher proportion of detectable orthopoxvirus-specific CD4 T cells than CD8 T cells 20–55 years after vaccination, consistent with this observation.^13^ Alternately, it is possible that CD8^+^ T cells tend to target more conserved epitopes shared among orthopoxviruses and thus are not influenced by prior vaccinia exposure.

Routine smallpox vaccination was stopped across the world in the 1970s. Since then, the frequency and size of mpox outbreaks have been increasing. PLWH remain at greatly increased risk for severe mpox in these outbreaks. Our work here characterizes humoral and cellular immunity among this population post-mpox, while demonstrating the persistent immune bias and boost conferred by prior VV vaccination. Overall, it will be important to continue monitoring post-infection immunity in this susceptible, high-risk population, as well as to adequately vaccinate groups at increased risk for exposure.

### Limitations of the study

Our cohort had some limitations, primarily stemming from the availability of sufficient T cells from mpox patients enrolled at a single site during a 6-month span. These limitations included a small sample size (*n* = 15 patients) with just one severe mpox case (precluding clinical associations), cross-sectional design utilizing just a single time point for each individual, mpox case samples being derived from clinics serving primarily HIV-positive individuals (thus narrowing our population to primarily PLWH), and a fully male and 93% African American cohort. We specifically chose to analyze only individuals living with HIV, given that this population is far more likely to develop severe mpox from clade IIb as compared to HIV-negative individuals. This allowed us to analyze T cell responses in detail in a group with cellular immunodeficiency, but both restricted our total sample size and also meant that we could not apply our conclusions to HIV negative individuals. Regardless, this is still one of the largest cohorts to include T cell ICS analysis on mpox survivors with HIV, highlighting the need for larger such studies in the future. It would be interesting to investigate antibody levels and T cell responses longitudinally to confirm the associations we derived between immune responses and the number of days post-symptoms based on the single time point. Such data assessing the durability of the mpox-specific immune response will be critical for guiding vaccination and other preventative strategies in future mpox epidemics.

### RESOURCE AVAILABILITY

#### Lead contact

Requests for further information and resources should be directed to and will be fulfilled by the lead contact, Rama R. Amara (ramara@emory.edu).

#### Materials availability

Modified vaccinia Ankara (MVA) was produced by standard methods and is additionally available in reasonable quantities from the Amara lab (Emory University) on request. Peptide pools for MPXV and conserved orthopoxviruses were produced by Professors Grifoni and Sette as described previously^34^ and available on request from them (agrifoni@lji.org and alex@lji.org).

#### Data and code availability

Data have been made publicly available at the Harvard Dataverse: https://dataverse.harvard.edu/dataverse/HIVmpox/. Shared data include numerical data for all figures as well as raw data output from Flowjo. Any additional information required to reanalyze the data reported in this paper is available upon request from the lead contact.

## STAR★METHODS

Detailed methods are provided in the online version of this paper and include the following:

### EXPERIMENTAL MODEL AND STUDY PARTICIPANT DETAILS

#### Human subjects and frozen specimens

Frozen serum and peripheral blood mononuclear cells (PBMCs) were used from people living with HIV who had donated specimens to the Emory Center for AIDS Research biorepository (Emory University, P30 AI050409). This biorepository stores frozen serum, tissue, and purified PBMCs from PLWH and healthy controls for use in ongoing and future studies. Serum was stored at −80°C and PBMCs were stored in liquid nitrogen. In this study, we used historical samples from 2010 to 2019 (mpox-negative) and post-mpox-infection samples from 2022 to 2023. This study was approved by the Emory Institutional Review Board (IRB00009146) as part of the Center for AIDS Research specimen repository study. Written informed consent was obtained from all participants. Sample acquisition protocols were compatible with the Declaration of Helsinki and donor information was anonymized. Demographics of patients and controls are listed in Table 1, with additional clinical data in Table S1. Socioeconomic status and ancestry were unavailable. The study was limited to available PBMC specimens from mpox patients and archived controls, resulting in all patients (and 70% of controls) being male, which may limit generalizability to all genders.

### METHOD DETAILS

#### PBMC thawing

Frozen PBMCs in 0.5–1 mL aliquots were thawed by heating in a 37°C water bath until only a sliver of ice remained, and then gently added to 8 mL pre-warmed Complete RPMI medium. Complete medium is RPMI-1640 medium (Fisher #MT-10-040-CVRF) supplemented with gentamicin at 10 μg/mL (Lonza 17518Z), 1% penicillin/streptomycin (Cellgro 30-002-C1), 10% FBS, and 1% HEPES buffer pH 6.98–7.3 (Lonza 17737E). Up to 3 aliquots from the same donor were used per tube. Samples were pelleted at 250 g for 7 to 15 min, the supernatant was removed by aspiration, then samples were incubated at 37°C in for 15 min in 20 units/mL DNAse I (Roche 4716728001) to reduce cell clumping. Samples were washed again with 10 mL complete medium, pelleted again at 250 g, and resuspended at 1–2 million cells/mL. They were then rested for 4–6 h at 37°C at a 10-degree angle before further use.

#### ICS stimulation preparation

Lyophilized peptide pools were resuspended in DMSO at 1 mg/mL of each peptide. Orthopoxvirus peptide pools (OpoxCD4, OpoxCD8, MpoxCD4, and MpoxCD8 pools 1 through 5^34^) were generously gifted by Dr. Alba Grifoni and Dr. Alessandro Sette (La Jolla Institute for Immunology). BEI resources provided peptide pools from HIV Gag (ARP-12425) and human cytomegalovirus pp65 (ARP-11549). Modified Vaccinia Ankara was produced and titered in our lab using standard techniques and used at MOI of 2.5–5.

#### ICS procedure

Samples were prepared at 1–2 million PBMCs/tube in 100 μL Complete RPMI. They were incubated for 15 min with 0.5 μg/mL of anti-CD40 (Miltenyi 130-094-133) for consistency with previous assays, and then combined to a final concentration of 1 μg/mL each of anti-CD28 (BD Pharmingen #555725) and anti-CD49d (BD #555501). Peptide pools were added to a final concentration of 1 μg/mL of each peptide, while MVA was used at 5 × 10^6^ million PFU (2.5–5 multiplicity of infection). DMSO alone was used in the non-stimulated sample (NS). Samples were incubated 12 h at 37°C before adding Brefeldin A (BD Golgiplug Cat #555029) and monensin (BD Golgistop Cat #554724) at 1:1000, along with anti-CD107a-BV786 (5 μL; BD #563869). Samples were incubated 4 more hours at 37°C, washed with FACS wash (2% FBS in PBS with 0.05% NaN_3_), and then surface stained with anti-CD8-BUV496 (2 μL; BD Horizon #612942), anti-CD4-BV650 (2 μL; custom conjugate), Live/dead (0.1 μL; APC-Cy7 channel, Invitrogen L34976) for 20 min at room temperature. Samples were washed with FACS wash and then fixed with 200 μL BD Cytofix/Cytoperm (Cat #554722) 20 min at 4°C. They were washed twice with perm wash (BD #554723), blocked using 10 μL human AB sera (Sigma H6914-20ML) for 15 min at room temperature, and then stained intracellularly for 25 min at 4°C. Intracellular stains included TNFα-PE-Texas red (2.5 μL; custom conjugate), IL-2-PE-Cy7 (2.5 μL; BD Pharmigen 560707), IFNγ-A700 (2.5 μL; BD/Fisher 557995), CD3-PerCP-Cy5.5 (2.5 μL; BD Pharmigen 552852), Perforin-BV421 (5 μL; Biolegend 353307), and IL-21-Alexa Fluor 647 (10 μL; BD Pharmigen 560493). IL-4-PE (2.5 μL; Miltenyi 130-123-698) and IL-17-FITC (2.5 μL; Thermo-Fisher 11-7179-42) were used in samples except those for CD8 analysis alone (thus, omitted for OpoxCD8 and MpoxCD8 pools 1–5). After staining, samples were washed with perm buffer, then FACS buffer, and then the entire sample tube was acquired on a BD LSR Fortessa flow cytometer using BD FACSDiva software version 9.0.

#### ICS data processing

Raw flow data was processed in Flowjo version 10.9. Gating was as described in Figure S1, using a time gate on each sample to remove the last couple seconds of data collection (which contain air that contributes to background). Cell counts and cytokine percent frequences were outputted in tabular format and processed further in Microsoft Excel 2019. A minimum of 5000 CD4 and CD8 cells were analyzed per sample, yielding a median of 68,180 (IQR 41,930–92,712) CD4 cells and 82,210 (IQR 39,501–109,616) CD8 cells. For all cytokines detected, the NS signal was subtracted from each stimulation. Only stimulation values that were at least double the NS (before NS signal subtraction) and corresponded to at least 5 cells (after NS signal subtraction) were considered positive; otherwise they were set at the lower limit of detection (0.001%). MpoxCD8 pools 1–5 each individually had the NS subtracted, were evaluated for true positivity as above, and then had only the positive signals combined to yield the final MpoxCD8 signal. For analyses of polyfunctionality only samples with at least 20 total positive cells were evaluated. SPICE^52^ software was used to group samples by polyfunctionality status. Statistical analyses were done using primarily Graphpad Prism 10 and occasionally JMP Pro 17 and Microsoft Excel 2019.

#### Whole virus ELISA

Clear flat-bottom 96-well plates (Nunc 88040LE from Thermo Scientific) were coated overnight at 4°C with 50 μL PBS containing either 5 × 10^6^ PFU/well of UV-inactivated MVA, 0.5 μL of gamma-irradiated clade IIb MPXV-infected BSC-40 cell lysate (BEI resources NR-59452), or 0.5 μL uninfected BSC-40 cell lysate. The next day, they were blocked for 2 h at room temperature with 5% nonfat dried milk (Bio-rad 1706404) dissolved in a 4% Whey buffer in PBST (containing 0.05% Tween 20). Primary antibodies dissolved in 4% Whey PBST were added with 3-fold dilutions and incubated 2 h at room temperature. Plates were then washed in PBS; HRP-conjugated goat-*anti*-human secondary antibody (SouthernBiotech 2045-05) was added at 1:10,000 in 4% Whey PBST and incubated for 1 h at room temperature. Plates were washed and then developed for 30 min using TMB (sera care 5120-0047); the reaction was stopped using 1 molar phosphoric acid and absorbance was read at 450 nm using a Varioskan Lux plate reader (Thermo VL0L00D2). MVA endpoint binding titers were calculating using 4-parameter logistical regression in Graphpad Prism 10 to determine the serum concentration that would yield a signal matching double the signal from MVA-coated wells containing 4% Whey PBST alone (no sera). For mpox binding titers, the ELISA signal from the uninfected cell lysate was subtracted from the corresponding serum dilution in the mpox-infected cell lysate; these modified values were used in 4-parameter logistical regression to determine endpoint titers.

#### MPXV and VV neutralization

The plaque reduction neutralization test (PRNT) was done as described previously.^42^ Briefly, serially-diluted serum was heat inactivated for 1 h, incubated with 50–80 PFU of clade IIb MPXV or VV, and then added to 96-well plates containing vero E6 monolayers. After 1 h of incubation at 37°C, wells were overlaid with 2% methylcellulose and incubated another 30 h at 37°C, and then inactivated and stained using crystal violet containing 10% buffered formalin. The Cellular Technology Limited Immunospot reader (S6 Micro Analyzer, ImmunoSpot) imaged the plates; plaques were counted using BioSpot software (Immunospot) and the PRNT_50_ values were calculated using 4-parameter logistic regression in GraphPad Prism version 8. The scientists performing these assays were blinded as to whether the sample came from an HIV-positive/mpox-positive patient versus a HIV-positive/mpox-negative control.

### QUANTIFICATION AND STATISTICAL ANALYSIS

Details for all statistical analyses are included in all figure legends. Graphpad Prism 10 was used for all statistical tests as well as binding ELISA logistic regression to determine titers. Graphpad 8 was used for calculating PRNT_50_ values as this was done in a different lab. Significance was defined using 2-tailed tests with *p*-values of <0.05.

## Supplementary Material

Table S1

Figures S1-S9

SUPPLEMENTAL INFORMATION

Supplemental information can be found online at https://doi.org/10.1016/j.celrep.2025.116501.

## Figures and Tables

**Table 1:** HIV+ study population

	mpox (*n* = 15)	Controls (HIV+, pre-2021) (*n* = 10)	

Median age in years (range)	42 (22–66)	30.5 (23–37)	*p* = 0.005
Male (%)	15 (100%)	7 (70%)	*p* = 0.05
African American (%)	14 (93%)	7 (70%)	*p* = 0.26
Other Races (%)	Hispanic 1 (7%)	Caucasian 3 (30%)	–
HIV-positive %	100%	100%	–
Median CD4 cells/mm^3^ (range)	432 (127–1385)	472.5 (208–1065)	*p* = 0.69
CD4 < 200 cells/mm^3^ (%)	2 (13%)	0 (0%)	*p* = 0.50
Median CD8 cells/mm^3^ (range)	668 (479–1821)	583 (557–1377)	*p* = 0.74
Median CD4/CD8 ratio (range)	0.6 (0.3–1.3)	0.8 (0.5–0.9)	*p* = 0.18
HIV viral load copies/mL (range)	<20 (<20–147,911)	<20 (<20–89,008)	*p* = 0.87
HIV viral load <100 copies/mL (%)	11 (73%)	6 (60%)	*p* = 0.38
Born before 1972 (%)	5 (33%)	0 (0%)	*p* = 0.06
Any smallpox vaccination (%)	7 (47%)	0 (0%)	*p* = 0.02
Vaccination during epidemic (%)	1 dose: 3 (20%) 2 doses: 1 (7%)	N/A	–

**KEY RESOURCES TABLE:** 

REAGENT or RESOURCE	SOURCE	IDENTIFIER

Antibodies		

Mouse anti-Human CD40	Miltenyi Biotec	Catalog #130-094-133; RRID:AB_10839704
Mouse anti-human CD28	BD Pharmingen	Catalog #555725; RRID:AB_396068
Mouse anti-human CD49d	BD	Catalog #555501; RRID:AB_2130052
Mouse anti-human CD107a-BV786	BD Biosciences	Cat# 563869, RRID:AB_2738458)
Mouse anti-human CD8-BUV496	BD Horizon	Catalog #612942, RRID:AB_2870223
Mouse anti-human CD4-BV650	Custom conjugate	N/A
Mouse anti-human TNF-alpha PE-Texas red	Custom conjugate	N/A
Rat anti-human IL-2 PE-Cy7	BD Biosciences	Cat# 560707, RRID:AB_1727542
Mouse anti-human IFN-gamma Alexa Fluor 700	BD Biosciences	Cat# 557995, RRID:AB_396977
Mouse anti-human perforin-BV421	BioLegend	Cat# 353307, RRID:AB_11149688
Mouse anti-human IL-21 Alexa Fluor 647	BD Biosciences	Cat# 560493, RRID:AB_1645421
Mouse anti-human IL-4-PE	Miltenyi Biotec	Cat# 130-123-698, RRID:AB_2905285
Mouse anti-human IL-17A FITC	Thermo Fisher Scientific	Cat# 11-7179-42, RRID:AB_10805390
Goat Anti-Human IgG HRP-conjugated	SouthernBiotech	Cat# 2045-05, RRID:AB_2795676

Bacterial and virus strains		

modified vaccinia Ankara	This study	N/A
Dryvax Vaccinia Virus	CDC	Priyamvada et al.^42^
Clade II MPXV	CDC	Priyamvada et al.^42^
Gamma-irradiated clade Ilb MPXV-infected BSC-40 cell lysate with uninfected BSC-40 control	BEI Resources	Cat# NR-59452

Biological samples		

PBMC samples	Emory CFAR	N/A
Serum samples	Emory CFAR	N/A

Chemicals, peptides, and recombinant proteins		

MPXV and OPXV peptide pools	LJI (Grifoni & Sette)	Grifoni et al.^34^
HIV Gag peptide pool	BEI resources	Cat# ARP-12425
CMV pp65 peptide pool	BEI resources	ARP-11549
Brefeldin A (Golgiplug)	BD Biosciences	Cat #555029
Monensin (GolgiStop)	BD Biosciences	Cat #554724
LIVE/DEAD^™^ Fixable Near-IR Dead Cell Stain	Invitrogen	Cat #L34976
Cytofix/Cytoperm	BD Biosciences	Cat# 554722
Perm/Wash Buffer	BD Biosciences	Cat# 554723
Human AB Sera	Sigma-Aldrich	Cat# H6914-20ML
Nonfat dried Milk	Bio-rad	Cat# 1706404
TMB (3,3’,5,5’-tetramethylbenzidine) 2-Component Microwell Peroxidase Substrate Kit	SeraCare	Cat# 5120-0047

Software and algorithms		

Graphpad Prism 10 and 8	Graphpad	RRID: SCR_002798
Flowjo v10.9.0	Flowjo	RRID:SCR_008520
Varioskan Lux Plate Reader	Thermo Fisher	VL0L00D2
Immunospot reader (S6 Micro Analyzer, ImmunoSpot)	Cellular Technology Limited	2001726
BioSpot software	Cellular Technology Limited (Immunospot)	Biospot

Deposited data		

Raw Data	This study	Harvard Dataverse:https://dataverse.harvard.edu/dataverse/HIVmpox/

## Highlights

- HIV+ mpox survivors had polyfunctional virus-specific T_H_1-type CD4 T cell responses
- CD8 T cell responses co-expressed perforin, indicative of cytolytic potential
- All had high levels of MPXV-binding antibodies and detectable neutralization
- Prior smallpox or mpox vaccination resulted in significantly higher antibody titers
