# Supplementary material for: Mpox-specific cellular and humoral immunity in mpox survivors living with HIV
Source: Cell Rep. Author manuscript; Available in PMC 2026 Feb 6. (PMC12880552; doi:10.1016/j.celrep.2025.116501)
Supplement: Figures S1-S9 [file NIHMS2129957-supplement-Figures_S1-S9.pdf]

Cell Reports, Volume *44*

Supplemental information

Mpox-specific cellular and humoral immunity in mpox survivors living with HIV

Samuel D. Stampfer, Lalita Priyamvada, Shainy Sambyal, Sailaja Gangadhara, Margaret Moriarty, Panayampalli S. Satheshkumar, Alba Grifoni, Alessandro Sette, Anandi N. Sheth, Colleen F. Kelley, and Rama R. Amara

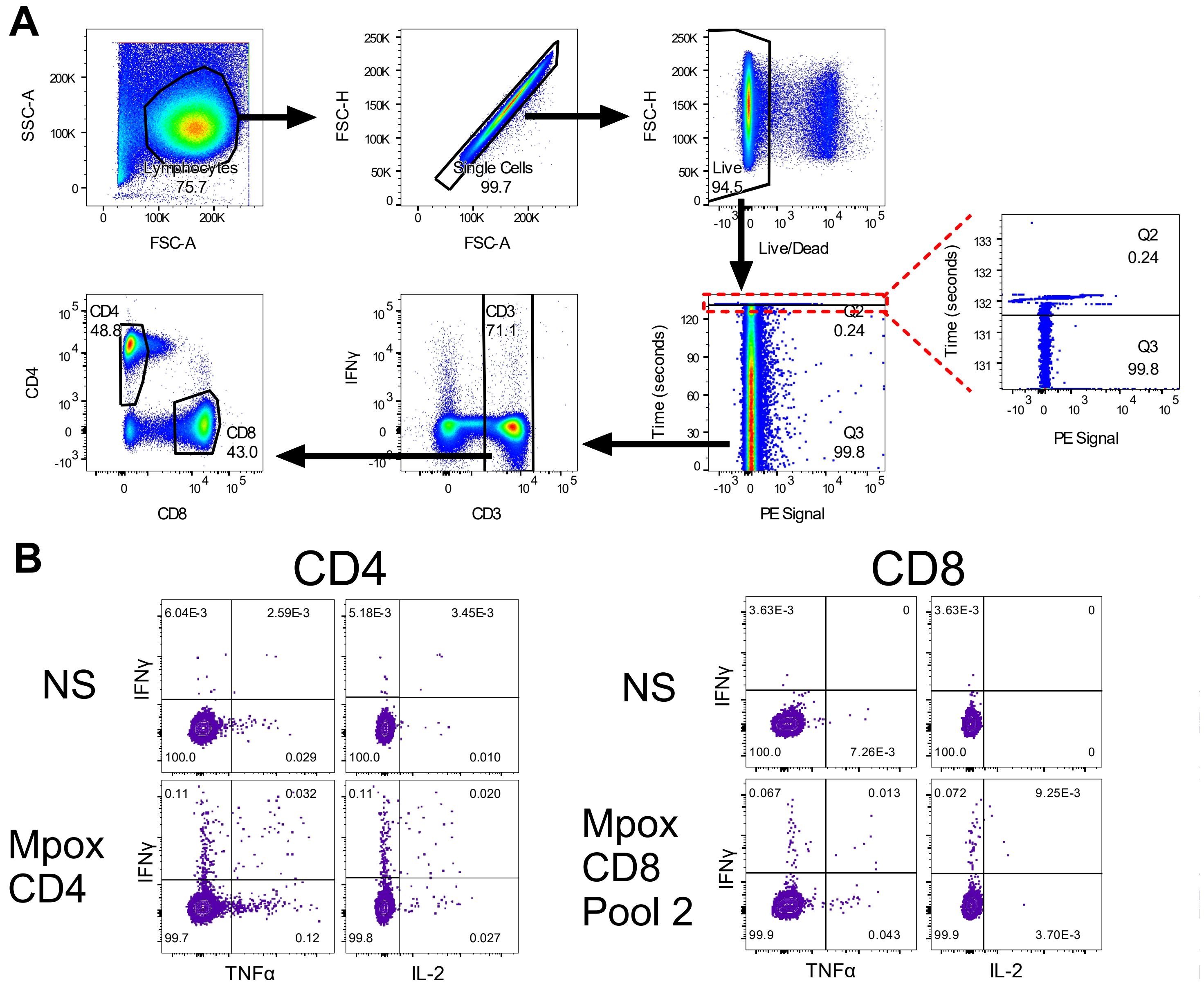

Fig S1. Gating strategy to improve detection of low ICS signals. Frozen cells were thawed and stimulated in ICS either as negative controls (NS, stimulated with DMSO only) or with the MpoxCD4 peptide pool (MPXV CD4-targeted).

A: Main gating strategy with exclusion of final high-background cells from each sample using a time gate.

B: Low-background ICS assay with sample that has low but detectable signal.

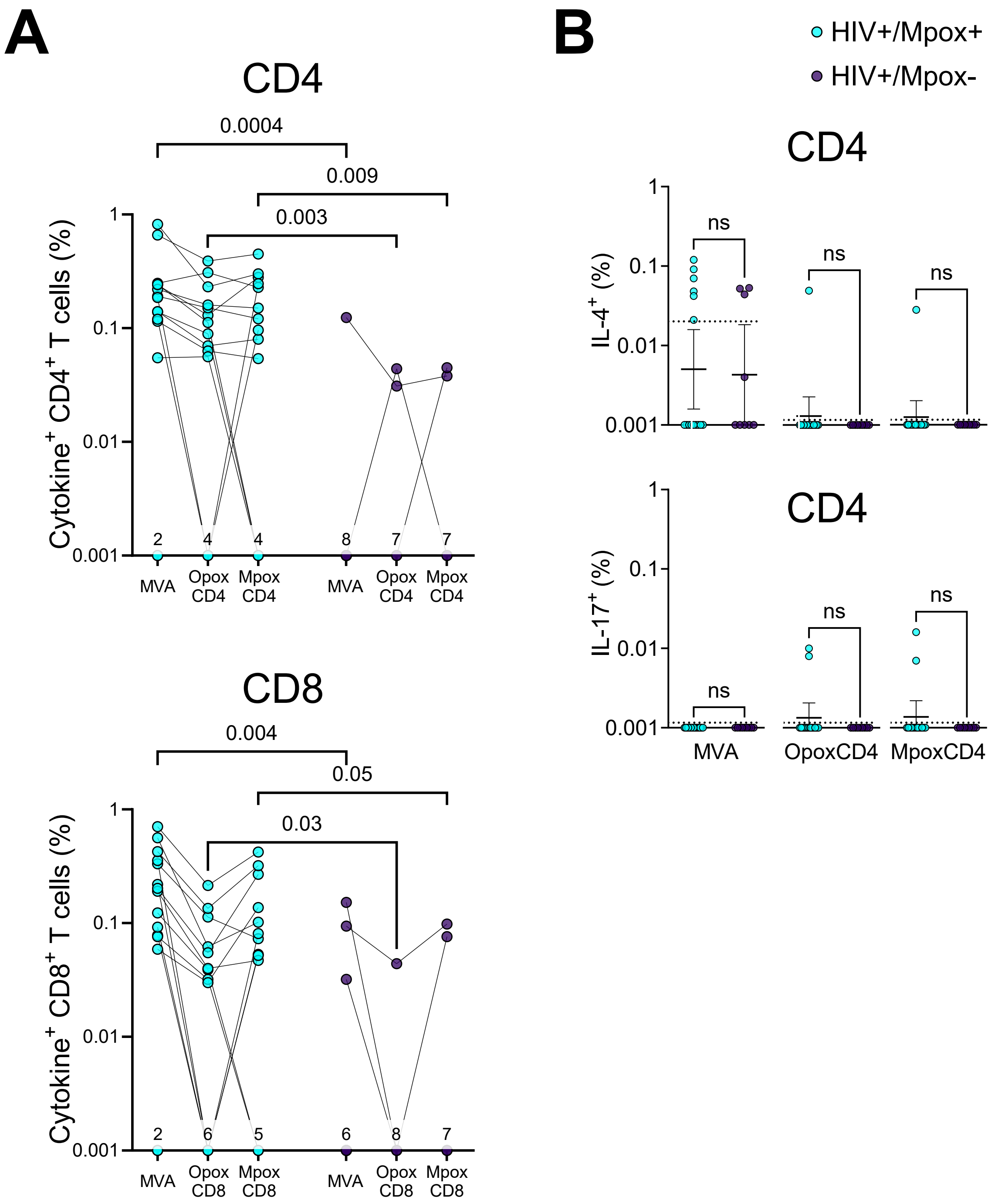

Fig S2. Additional ICS comparisons between patients and controls. ICS was done on PBMCs as described in Figure 1.

A: Total cytokine positive T-cells are indicated by combining signals for IFNγ, TNFα, and IL-2 positive cells using a Boolean "OR" test to avoid duplication. Lines connect the values for the same individuals with different stimuli. The combination increased the NS signal (background), resulting in additional patients whose poxvirus stimulation was less than double background and thus listed at the minimum (indicated by dots on the axis, with the number of subjects listed directly above the dot on the axis).

B: Total expression of IL-4 (top) and IL-17 (bottom) was calculated as a percent of total CD4+ T cells. Data are represented by the geometric mean (solid line) and its 95% confidence interval (error bars). Dotted lines indicate the 90th percentile cutoff of controls. Mann-Whitney tests were used to compare HIV-positive mpox-positive patients versus HIV-positive mpox-negative controls.

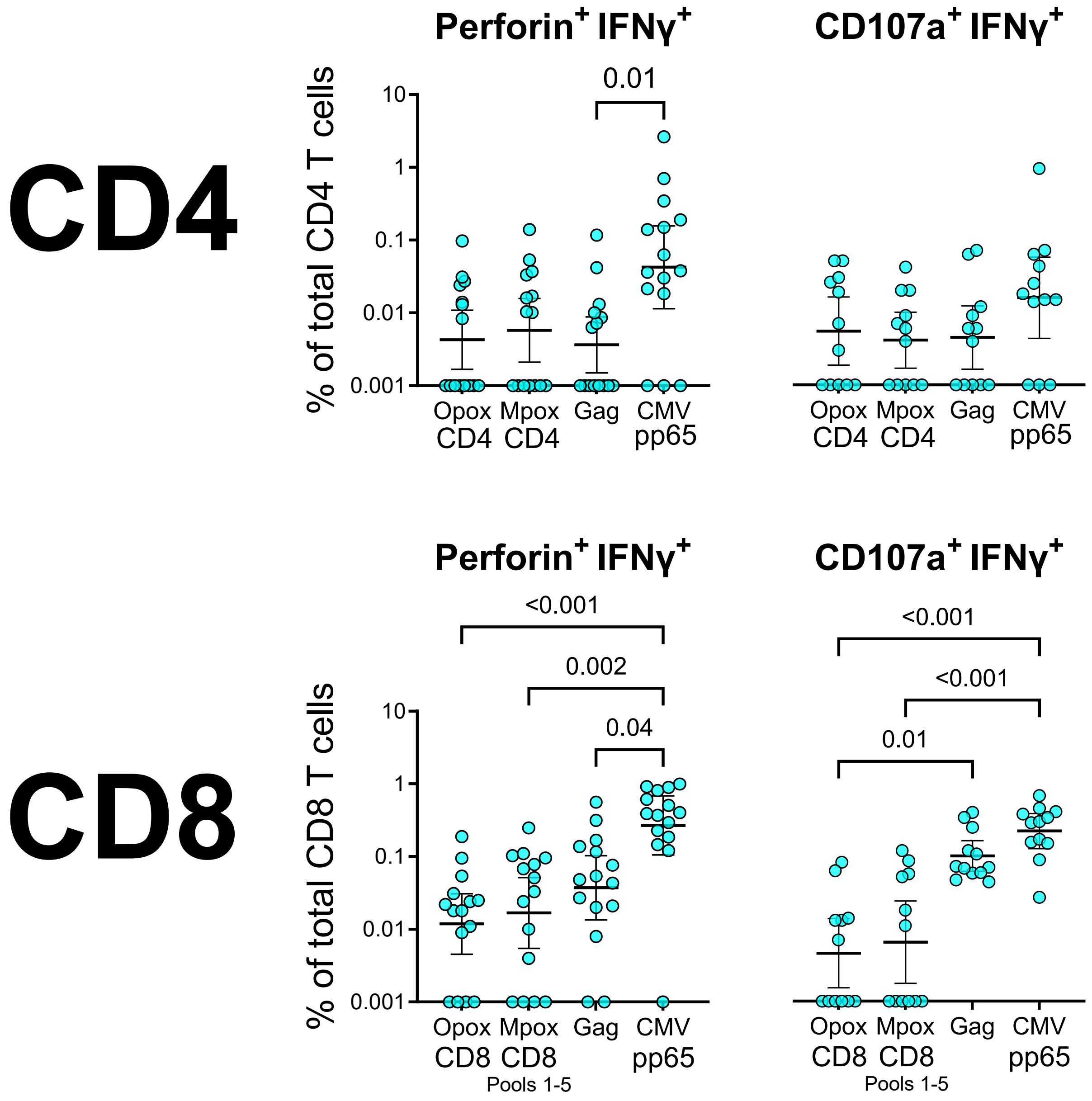

Fig S3. Evaluation of the absolute number of CD4 and CD8 IFNγ+ cells with co-expression of either Perforin or CD107a, as a percent of total CD4 or CD8 T cells. ICS was done on PBMCs as described in Figure 1. Total expression of IFNγ+ Perforin+ or IFNγ+ CD107a+ cells as a percent of total CD4+ (top) or CD8+ (bottom) T cells. p-values were calculated using matched data with nonparametric tests. Data are represented by the geometric mean and its 95% confidence interval. Multiple comparisons were corrected using Dunn's test.

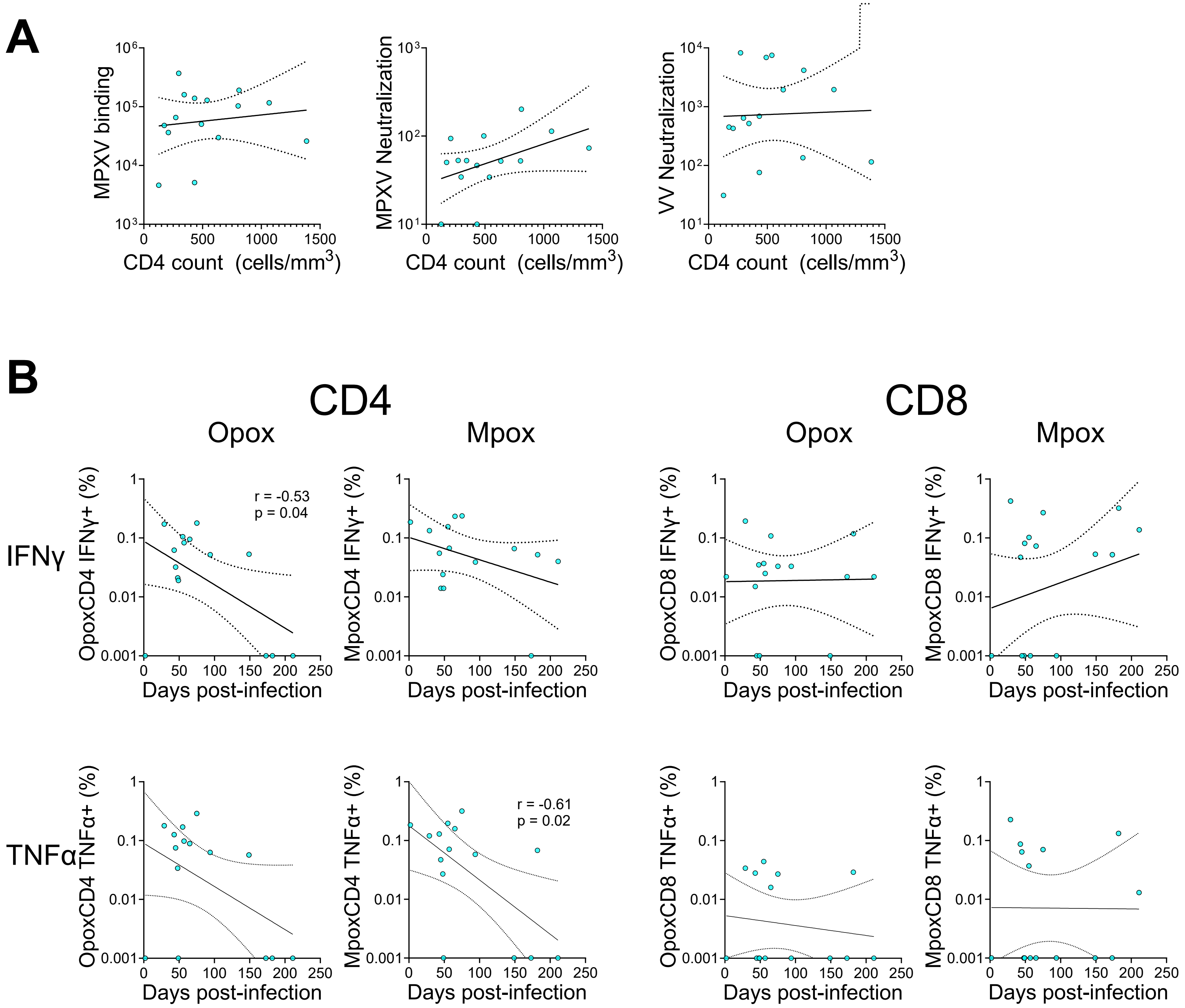

Figure S4. Correlations of immune responses with CD4 counts and sampling date.

A: Serologic samples from PLWH with recent mpox (n = 15) were tested in a binding ELISA assay against inactivated MPXV and in neutralization assays against live clade IIb MPXV and VV. Endpoint titers of the mpox patients were log transformed and correlated by linear regression against CD4 T cell count around time of mpox diagnosis. Data are represented by the linear regression best-fit line and its 95% confidence interval. Results were not statistically significant. **B:** Days from first symptoms were correlated by linear regression with the log-transform of T cell ICS signals. Data are represented by the linear regression best-fit line and its 95% confidence interval. Only significant comparisons are shown numerically. We did not correct for multiple comparisons. See also Fig 4E.

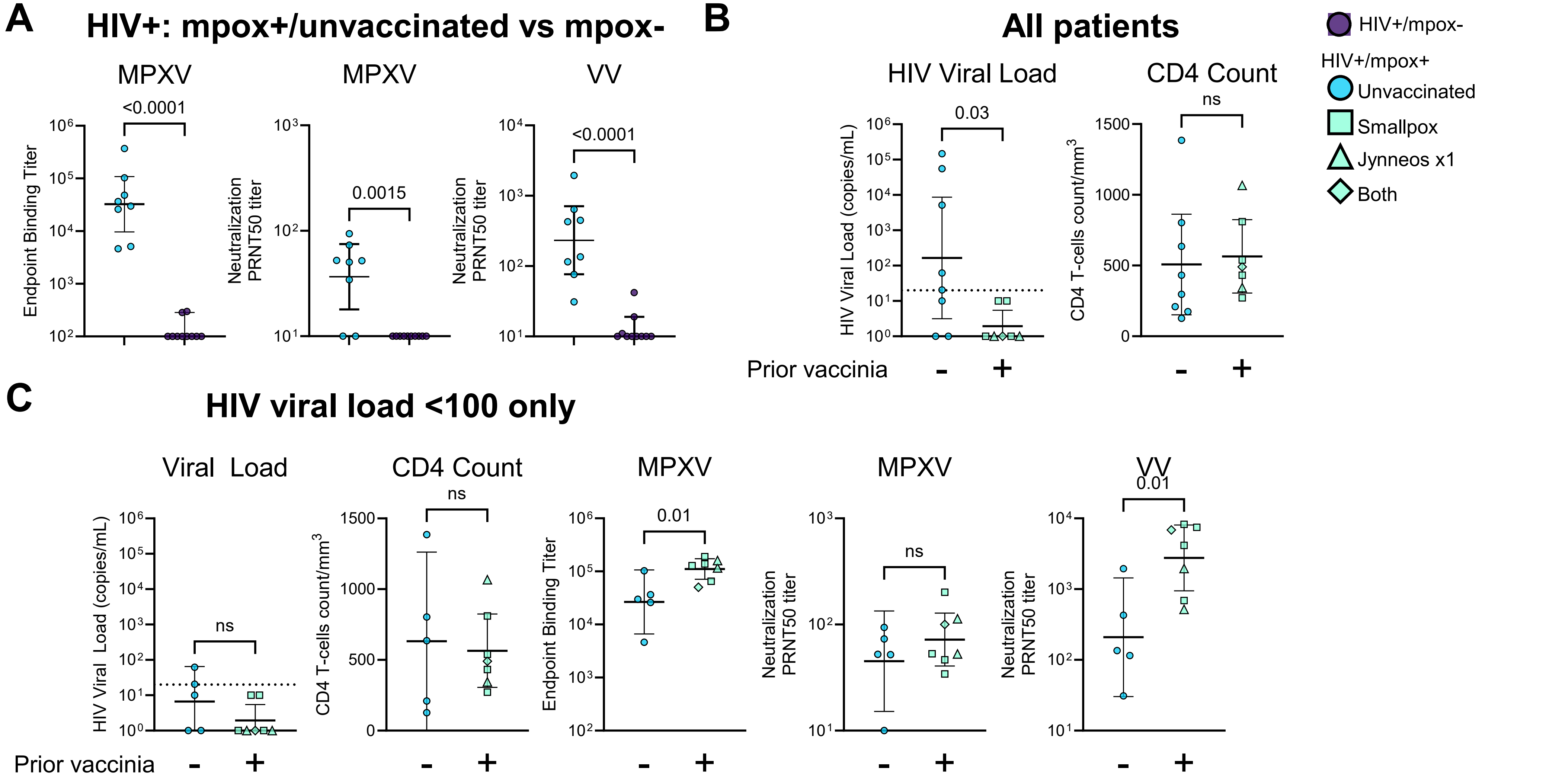

Fig S5. Humoral comparison between vaccinated and unvaccinated mpox survivors after controlling for HIV viral load. Eight mpox-survivors had no prior smallpox vaccination (circles), while three had historical pre-1972 smallpox vaccination (squares), two had a single dose of JYNNEOS MVA vaccine (triangles; administered shortly before patients contracted mpox), and two had both pre-1972 smallpox vaccination and JYNNEOS (diamonds). **A:** Comparison of humoral immunity of the eight HIV+ mpox survivors with no prior VV exposures, versus ten mpox-negative HIV-positive control individuals who had never received smallpox vaccination. Data are represented as the geometric mean and its 95% confidence interval. **B:** CD4 count and HIV viral load comparison between vaccinated and unvaccinated. Viral load limit of quantification (dotted line) was 20 copies/mL. For logarithmic analysis purposes (where no value can be equal to zero), detectable but unquantifiable viral loads <20 were set as 10 copies/mL and undetectable viral loads were set as 1 copy/mL. Data are represented as the geometric mean (for HIV viral load) and mean (for CD4 count) with error bars representing the 95% confidence interval. **C:** CD4 count, HIV viral load, and binding and neutralizing antibody comparisons in the subset of patients with HIV viral load <100 (includes five unvaccinated individuals and all seven vaccinated individuals). All pairwise comparisons were via the nonparametric Mann-Whitney T-test. Data are represented as the geometric means and 95% confidence interval for all parameters except CD4 count, which is displayed as the mean and 95% confidence interval.

Historical smallpox vaccinees vs unvaccinated

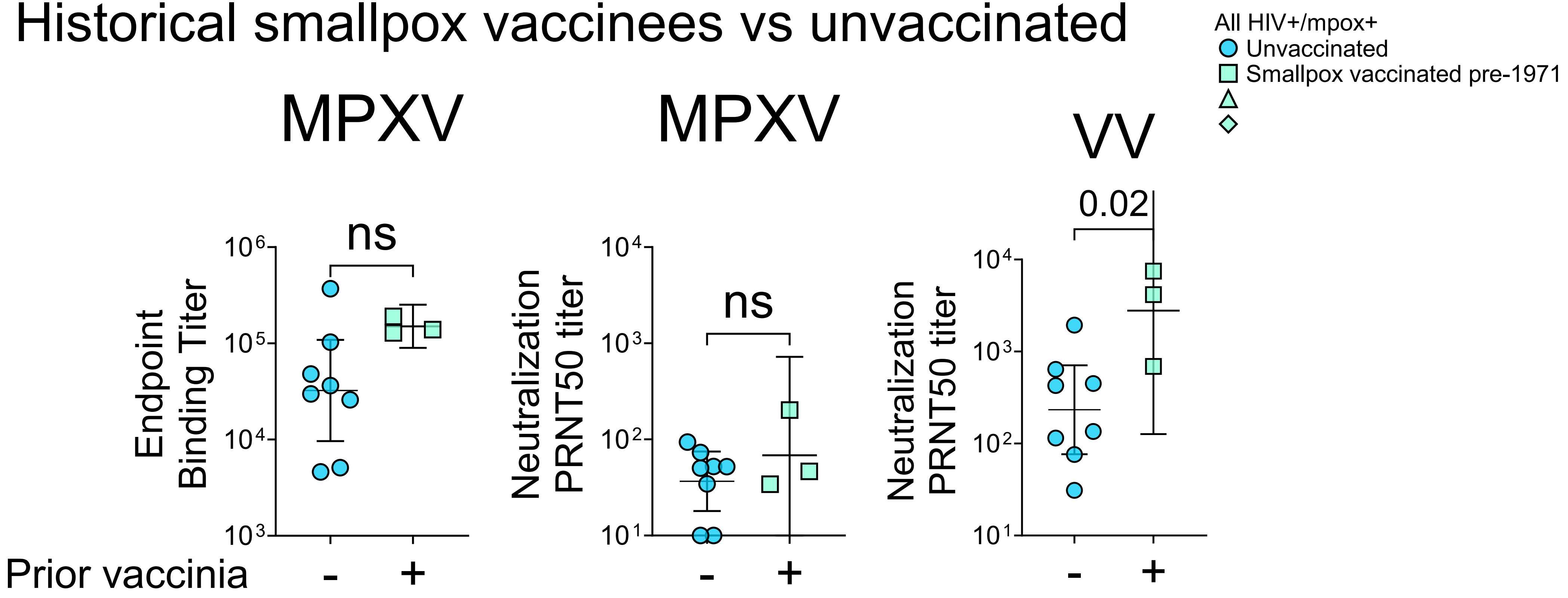

Fig S6. Humoral data in mpox patients with no smallpox or mpox vaccination versus those vaccinated pre-1971. Serologic samples from PLWH with recent mpox. One group (circles) had no prior mpox or smallpox vaccination (n = 8); the other group (squares; n = 3) were presumed vaccinated for smallpox in childhood based on birthdate before 1970 and did not have any subsequent smallpox or mpox vaccination. The binding ELISA assays was done against inactivated MPXV; neutralization assays were done against live clade IIb MPXV or VV. Pairwise comparisons were via the nonparametric Mann-Whitney t-test; data are represented as the geometric mean and its 95% confidence interval.

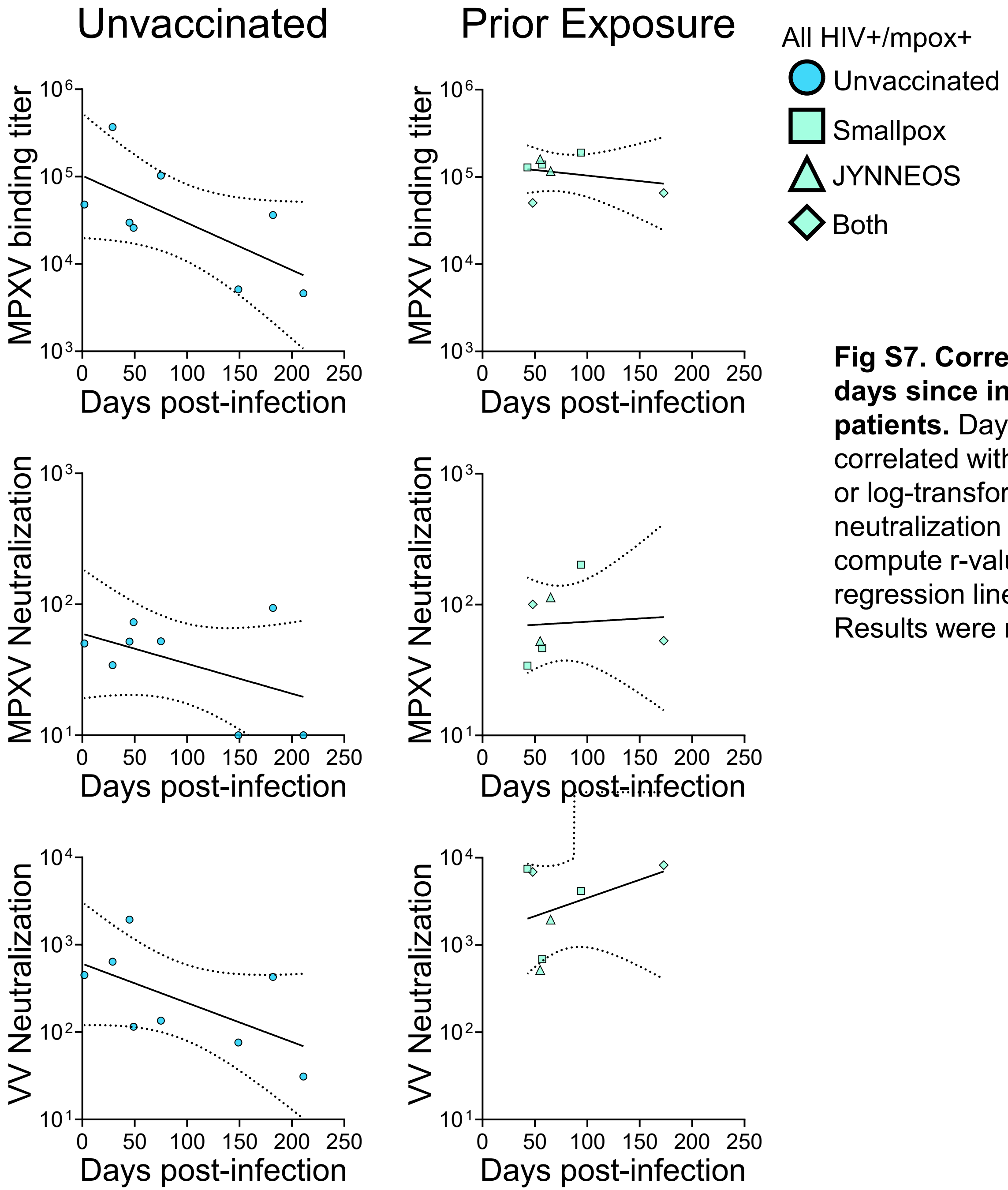

Fig S7. Correlation between antibody titer and days since infection in unvaccinated vs vaccinated patients. Days since initial symptom onset were correlated with log-transformed endpoint binding titers or log-transformed MPXV and VV PRNT50 neutralization titers. Linear regression was used to compute r-values. Data are represented by the linear regression line and its 95% confidence interval. Results were not statistically significant.

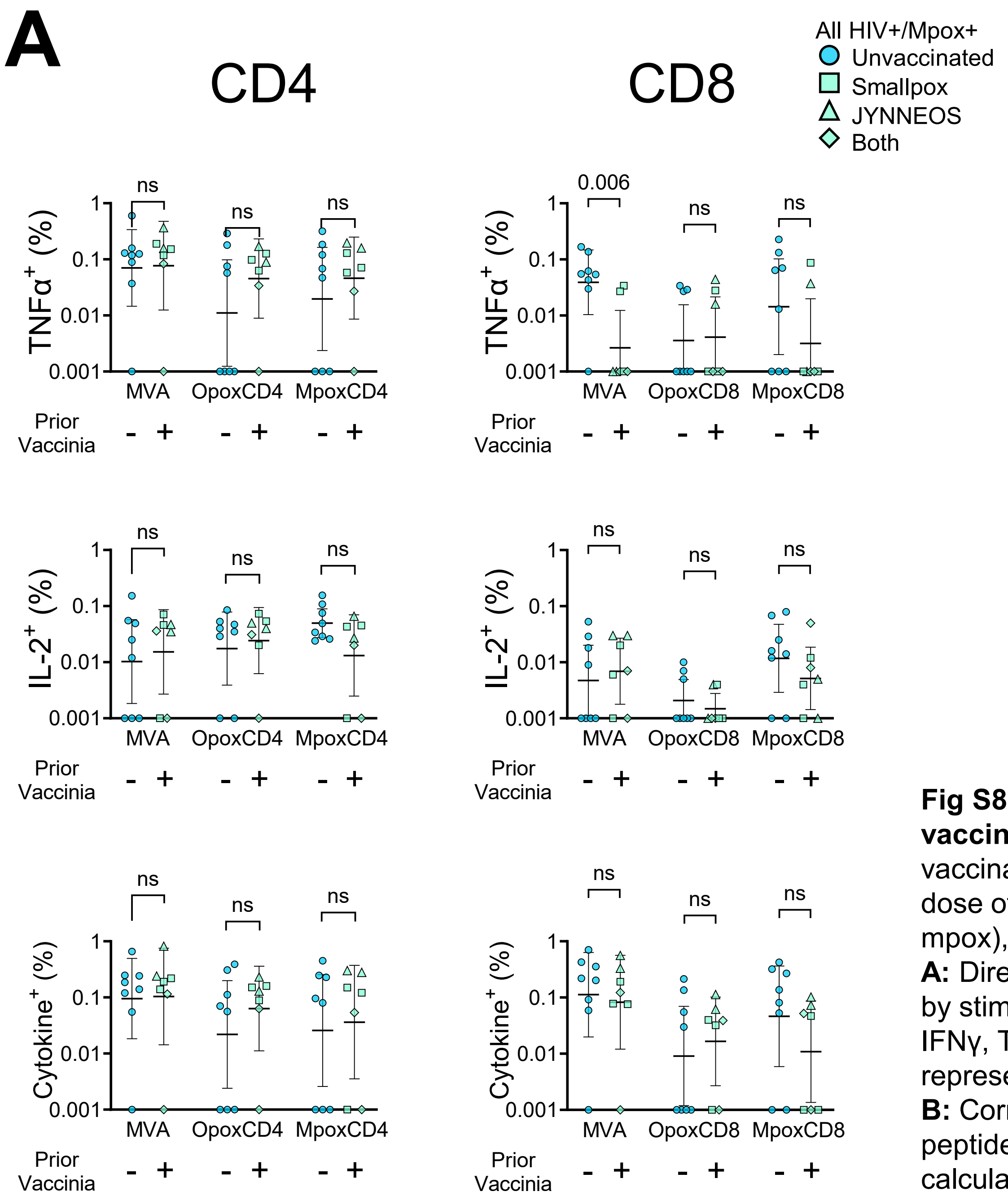

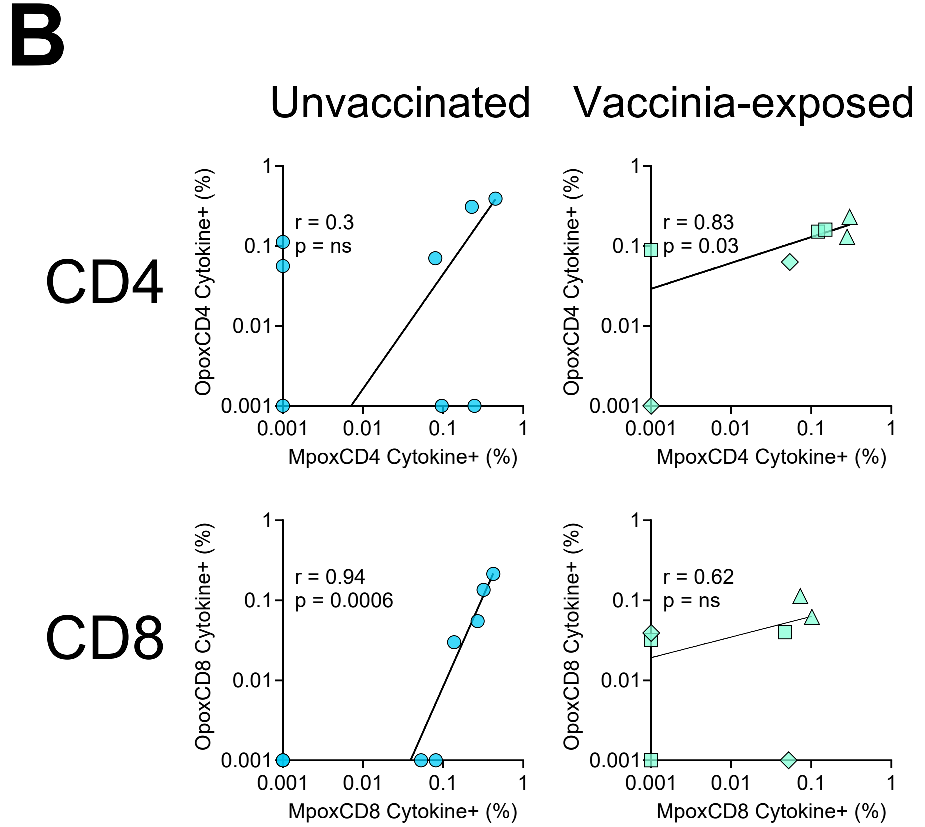

Fig S8. Comparison of orthopoxvirus-specific TNFα, IL-2, and total cytokine production by vaccination status. ICS was done on frozen cells. Eight mpox-survivors had no prior smallpox vaccination, while three had historical pre-1972 smallpox vaccination (squares), two had a single dose of JYNNEOS MVA vaccine (triangles; administered shortly before patients contracted mpox), and two received both smallpox and JYNNEOS vaccinations (diamonds).

A: Direct comparison of TNFα, IL-2, and total cytokine signal for CD4 and CD8 T-cells grouped by stimulation type (MVA virus or peptide pools). Total cytokine is the Boolean combination of IFNγ, TNFα, and IL-2 via "OR" gates. P-values calculated using Mann-Whitney t-tests. Data are represented by the geometric mean and its 95% confidence interval.

B: Correlation of T-cell responses to MPXV-specific vs cross-reactive orthopoxvirus-specific peptide pools in CD4 (top) and CD8 (bottom) T-cells. r-values and p-values (95% two-tailed) were calculated using the nonparametric Spearman test. The best fit lines were placed using logarithmic nonlinear regression. See also Figures 5B and 5C.

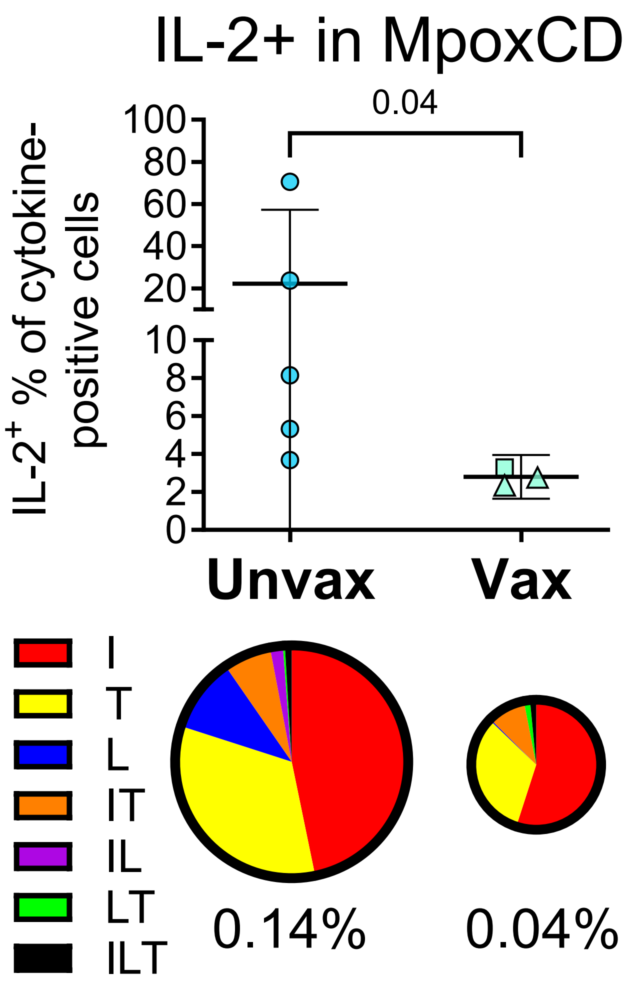

Fig S9. CD8 IL-2 and polyfunctionality comparison in based on smallpox/mpox vaccination status. ICS was done on PBMCs using the MCD8 peptide pools with total signals combined. Top: IL-2 percent positivity as a proportion of total cytokine-positive cells (comparison by Mann-Whitney); data are represented by the mean and its 95% confidence interval. Bottom: Proportions of cells positive for all combinations of IFNγ (I), TNFα (T), and IL-2 (L), with pie charts based on the geomeans for each type. Total signal is indicated underneath each pie chart. Only samples with at least 20 cytokine-positive cells were included in polyfunctionality analyses, but total signal magnitude included all samples.
